# Sono‐Anatomy and Histology of the Deep Fasciae in the Upper Limb

**DOI:** 10.1002/jum.70292

**Published:** 2026-05-13

**Authors:** Carmelo Pirri, Nina Pirri, Lucia Petrelli, Veronica Macchi, Andrea Porzionato, Raffaele De Caro, Carla Stecco, Levent Özçakar

**Affiliations:** ^1^ Department of Neurosciences, Institute of Human Anatomy University of Padova Padova Italy; ^2^ Department of Medicine—DIMED, School of Radiology, Radiology Institute University of Padua Padova Italy; ^3^ Department of Physical and Rehabilitation Medicine Hacettepe University Medical School Ankara Turkey

**Keywords:** arm, deep fascia, finger, forearm, hand, shoulder, ultrasonography

## Abstract

The deep fascia of the upper limb represents a pivotal anatomical structure essential for effective force transmission, dynamic compartmentalization and musculoskeletal stability. Its composition (rich in type I collagen fibers) enables both mechanical resilience and functional adaptability, crucial for the upper limb's complex movements. Recent advancements in high‐resolution ultrasonography, complemented by anatomical/histological studies, have provided unprecedented insights into the fascia's microarchitecture and clinical significance. This paper delivers a simple and systematic guide as regards the sono‐anatomy of deep fasciae in the upper limbs, including brachial, antebrachial and palmar fasciae as well as their continuity with adjacent structures. Integrating anatomical, histological and sonographic evidence, this article sheds light on the clinical relevance of fascia‐centric approaches applied in rehabilitative and surgical treatments.

**Abbreviations**
APL: abductor pollicis longus
ECRB: extensor carpi radialis brevis
ECRL: extensor carpi radialis longus
ECU: extensor carpi ulnaris
ED: extensor digitorum
FCR: flexor carpi radialis
FCU: flexor carpi ulnaris
FDP: flexor digitorum profundus
FDS: flexor digitorum superficialis
MCP: metacarpophalangeal
US: ultrasound

Recent advancements in high‐resolution ultrasonography (US) have revolutionized the study of deep fascia by providing non‐invasive, real‐time visualization of its structure and function.^1^ US examination not only delineates its layers but also captures dynamic interactions, such as gliding between fascial planes and muscle contractions.^2^ Moreover, US aids in diagnosing pathological conditions, including adhesions, fibrosis, fascial tears, etc., while offering valuable/close follow up during rehabilitation. Accordingly, US appears to be an indispensable tool for integrating anatomical knowledge with clinical practice, enabling precise diagnosis and targeted therapeutic interventions.^3^

The deep/muscular fascia, a dense connective tissue, envelops and separates muscles, vessels, and nerves, serving as a crucial structural and functional framework. Its role in the upper limb extends beyond compartmentalization to include dynamic contributions to force transmission and biomechanical efficiency.^4^ The intricate architecture of the deep fascia, characterized by collagen fibers, ensures both mechanical stability and adaptability, essential for coordinated movement throughout the body, including the upper limb's range of motion.^5^

To the best knowledge of the authors, there is no atlas fusing the US, anatomical and microscopic images that can be used for relevant clinical understanding. Therefore, proposing a contemporary approach, this article aims to integrate these findings to elucidate the regional anatomy of deep/muscular fascia in the upper limb.

## Methodology

To construct a solid anatomical reference, images were derived from 10 cadaveric specimens using standard sectional planes stained with hematoxylin and eosin. The human body donors had signed a document agreeing to participate in the body donation program of the medical school—Institute of Human Anatomy of the University of Padova. This study was conducted in accordance with the principles of the Declaration of Helsinki. Every effort was made to follow local/international ethical guidelines and laws that pertain to the use of human cadaveric donors in anatomical research.^6^

To establish a correlation between anatomical and US images, we obtained sonographic images from 10 volunteers, mapping anatomical and histological features directly onto scans. The annotation process was validated through consensus among 5 expert sonographers. Any discrepancies in annotations were resolved collaboratively, ensuring adherence to the official literature on fascial US imaging. Image acquisition was performed using an Aplio i800 CANON (with i24LX8, i22LH8, i18LX5, and 14L5 probes). Using a proximal to distal approach, systematic illustrations of anatomy and US will be combined/provided with clinical relevance in the rest of this paper. US examination of the deep fascia of the upper limbs is a crucial tool for assessing its structural integrity, mobility and pathological changes, offering high‐resolution and real‐time visualization. The deep fascia appears as a hyperechoic linear structure with alternating hypoechoic sublayers that envelops muscles,^1^ acting as a key element in force transmission and compartmentalization. High‐frequency linear transducers with a total range of 5–24 MHz provide superior resolution according to the depth, location of deep fascia and BMI patients.^1^ Proper US imaging parameters, including adjusted gain, dynamic range and optimal focus placement, enhance clarity and minimize artifacts like anisotropy—caused by collagen fiber orientation—while ensuring the US depth is set for clear fascial layer visualization. The probe is positioned perpendicular to the fascial planes to prevent anisotropy artifacts, adequate gel is applied to avoid excessive compression that could alter fascial thickness, and both longitudinal and transverse scans are systematically correlated for a comprehensive assessment.

Normal deep fascia exhibits smooth gliding movement over muscles during dynamic scanning, whereas fascial adhesions, fibrosis, densification or thickening appear as hyperechoic/hypoechoic zones with restricted mobility. Fluid collections or inflammation within or near the deep fascia appear as hypoechoic or anechoic regions, which may indicate fasciitis or post‐traumatic edema. Power and Color Doppler are used to detect vascular elements or changes within or near the deep fascia of the upper limb, particularly in inflammatory conditions such as compartment syndromes or fasciitis, where increased blood flow suggests active pathology. Shear wave elastography (SWE) provides valuable information for detecting fibrosis, myofascial pain syndromes and post‐surgical adhesions. Increased stiffness on elastography is correlated with restricted movement and chronic fascial dysfunction such as overuse injuries. Real‐time dynamic US examination evaluates the gliding function of deep fascia in cases of chronic pain or fascial nerve entrapments where mobility may be restricted. Sono‐palpation is used to assess fascial compliance, differentiating between fluid‐filled spaces, fibrotic bands and adhesions. The integration of advanced sonographic techniques, including elastography and Doppler, enhances diagnostic precision, facilitating early intervention and personalized treatment strategies for deep fascia‐related musculoskeletal disorders. Moreover, US examination is essential for guiding myofascial interventions like fascial plane blocks, hydrorelease and hydrodissection, ensuring precision, safety and efficacy. Finally, US examination is crucial to monitor fascial changes post‐surgery or trauma and evaluation of compartment syndrome risks.

## Shoulder

### Deltoid Fascia

#### Anatomy

**Figure 1:**
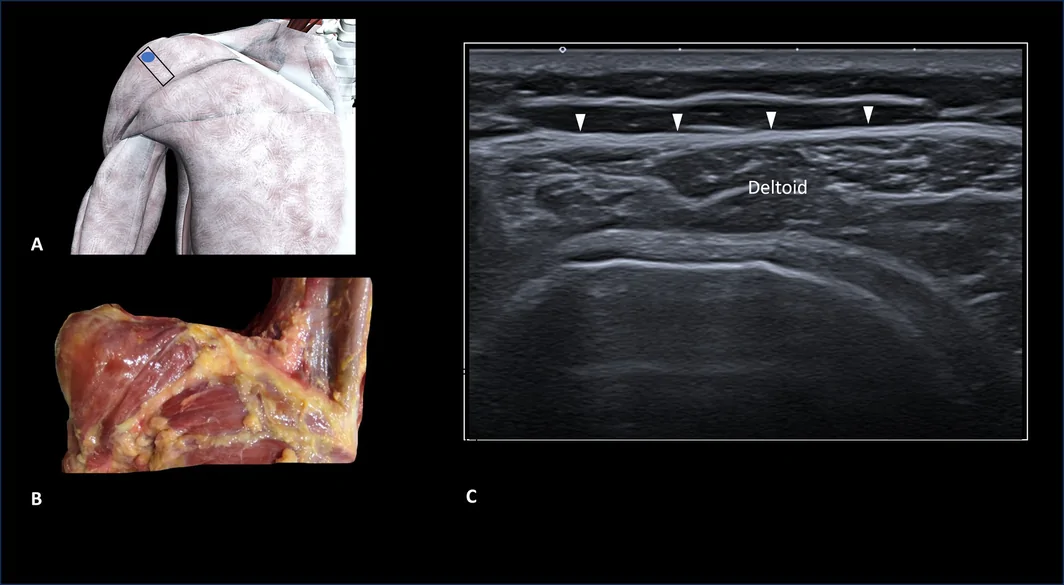
Deltoid fascia. **A**, Probe positioning. **B**, Anatomical dissection showing the deltoid fascia as a dense fibrous layer enveloping the deltoid muscle, continuous with the clavipectoral fascia proximally and the brachial fascia distally. **C**, Ultrasound image (*transverse view*) displaying the deltoid fascia as a hyperechoic linear structure superficial to the deltoid muscle, with well‐defined fascial‐muscular interface. Arrowheads: deltoid fascia.

The deltoid fascia exhibits significant variability in thickness among individuals, independent of the size of the underlying deltoid muscle (Figure 1).^7^ It adheres strongly to the muscle and provides structural continuity among its anterior, lateral and posterior portions, extending seamlessly into the brachial fascia.^7^ Proximally, it merges with the fascia covering the trapezius muscle. Overlying the acromion, scapular spine and clavicle, the deltoid fascia demonstrates partial adherence to the periosteum, contributing to force transmission and muscular compartmentalization.^8^ Histologically, it is composed of undulating collagen fibers arranged primarily in a transverse orientation relative to the underlying muscle.^8^ Almost 15% of its fibers are elastic and, with an irregular mesh‐like network, they provide biomechanical stability.^8^

#### Ultrasonography

High‐frequency US imaging reveals the deltoid fascia as a hyperechoic linear structure overlying the deltoid muscle. In addition to static evaluation, dynamic scanning can be performed during various shoulder motions to assess fascia gliding, elasticity, and interactions with the underlying tissues^9^, ^10^ (Figure 1).

#### Clinical Relevance

Pathologies such as adhesions, fluid collections, localized thickening, fibrosis, adhesive capsulitis, or scarring after trauma, inflammatory conditions, or surgical procedures can significantly limit shoulder function.^11^ US examination assists in diagnosing these conditions, guiding treatments such as targeted fascial release, hydrodissection (in the fascial plane near the functional joint of shoulder), and physical therapy. Evaluation of its relationship with the axillary nerve would be noteworthy in entrapment syndromes (Table 1).^12^

**Table 1:** Deep Fasciae of the Shoulder and Axillary Regions: Integrated Anatomical, Sonographic, and Clinical Features

Type of Deep Fascia	Average/Range Thickness	Sonographic Landmarks	Ultrasound Appearance	Clinical/Functional Relevance
Deltoid fascia	Variable; independent from deltoid muscle mass. Elastic fibers ≈15%	Over deltoid muscle; acromion, scapular spine, clavicle	Continuous hyperechoic line overlying deltoid; dynamic scanning during abduction or rotation highlights fascial gliding and elasticity	Fascial fibrosis, adhesions, post‐surgical scarring and inflammation may limit shoulder mobility. US guides hydrodissection, fascial release, and evaluation of axillary nerve entrapment
Axillary fascia	Two distinct laminae; thickness varies with adiposity and gender	Between pectoralis major and latissimus dorsi (superficial layer); between pectoralis minor and coracobrachialis (deep layer); neurovascular bundle as key reference	Two parallel hyperechoic bands beneath subcutaneous fat, enclosing axillary fat pad and neurovascular structures; best visualized with arm abducted and externally rotated	Critical in lymphatic drainage; damage leads to axillary web syndrome or shoulder dysfunction. US assesses fascial continuity post‐mastectomy and guides manual/fascial therapy
Subscapular fascia	<0.5 mm	Coracoid process, subscapularis and serratus anterior interface, axillary vessels	Thin hyperechoic sheet enveloping subscapularis, separating it from serratus anterior; best seen with arm externally rotated	Adhesions or fibrosis linked to frozen shoulder and scapulothoracic dysfunction; US detects fascial thickening and loss of gliding, guiding hydro‐release and rehab protocols
Infraspinatus fascia	Variable, up to 1 mm	Spine of scapula; infraspinatus and teres minor fossa; teres minor nerve course (~44 mm from insertion)	Hyperechoic lamina superficial to infraspinatus; assessed in short‐axis (parallel to scapular spine) and long‐axis (along fibers)	Fascial sling may entrap teres minor nerve (≈3% of shoulder pain cases). US discriminates fascial restriction from rotator cuff tears, monitors post‐rehabilitation fascial mobility
Supraspinatus fascia	~0.7 mm on average; thickening at spinoglenoid notch common	Scapular spine, supraspinatus fossa, greater tubercle; spinoglenoid notch	Continuous thin hyperechoic layer over supraspinatus; thickening with increased echogenicity suggests fibrosis. Dynamic scanning during abduction/external rotation evaluates gliding	Fascial thickening may compress suprascapular nerve even in absence of ligament. US identifies fascial entrapment, differentiates myofascial pain from tendon pathology, and guides perineural hydrodissection

### Axillary Fascia

#### Anatomy

**Figure 2:**
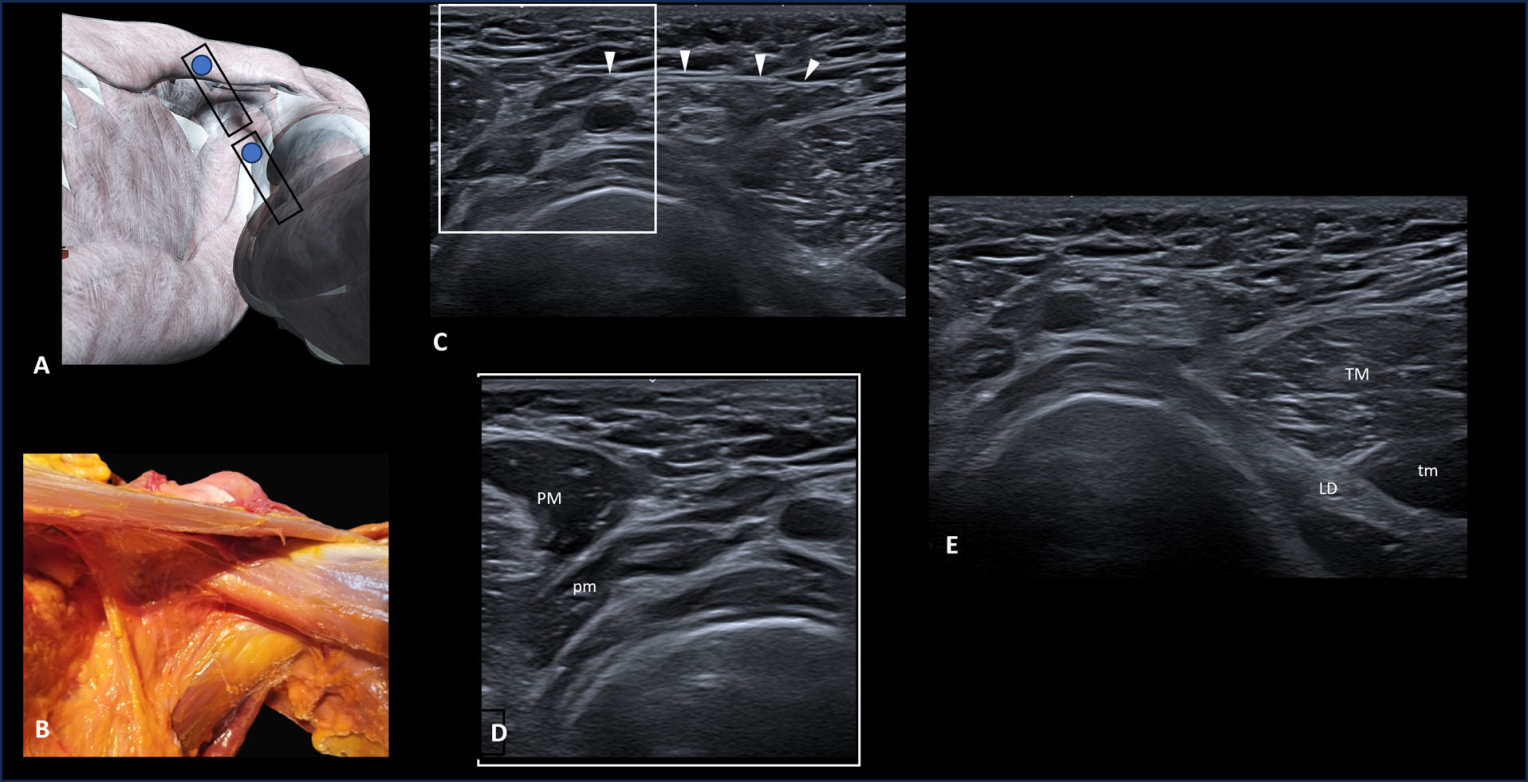
Axillary fascia. **A**, Probe positioning. **B**, Anatomical dissection showing the axillary fascia forming the floor of the axilla, extending between the pectoral and latissimus dorsi margins. Note its continuity with the brachial and clavipectoral fasciae and the presence of neurovascular perforations. **C**–**E**, Ultrasound images revealing the axillary fascia (arrowheads) as a thin, hyperechoic band separating the subcutaneous tissue from the deeper axillary contents, with adjacent visualization of lymph nodes and vascular elements. PM, pectoralis major muscle; pm, pectoralis minor muscle; TM, teres major muscle; tm, teres minor muscle; LD, latissimus dorsi muscle.

The axillary fascia is a robust fibrous structure, made up of 2 layers.^8^, ^13^ The superficial layer extends laterally to merge with the brachial fascia, medially and anteriorly with the pectoralis major fascia and posteriorly with the fascia of the latissimus dorsi muscle.^8^, ^14^ The deep layer extends from serratus anterior fascia, pectoralis minor fascia, and brachial fascia at the coracobrachialis muscle.^8^, ^14^ This fascial continuity reinforces the structural framework of the axillary region, facilitating dynamic interactions among the muscles that insert into and exert tension upon it.^15^, ^16^ Internally, the axillary fascia is intimately associated with the suspensory ligament of Gerdy, which serves as a critical linkage between the clavicopectoral and subscapularis fasciae.^8^ This anatomical relationship establishes a functional connection between the deep fascial layers of the scapular girdle, allowing for coordinate biomechanical responses during upper limb movements. Histological and anatomical studies reveal that multiple force transmission vectors can be discerned within the axillary fascia, reflecting diverse mechanical influences exerted by the surrounding musculature.^8^, ^13^ This intricate fascial network enables efficient distribution of mechanical loads, optimizing shoulder and arm kinematics while preserving the structural integrity of the axillary space (Figure 2).

#### Ultrasonography

The axillary fascia is identified as 2 hyperechoic lines beneath the subcutaneous tissue in the axillary fossa, the first layer between the pectoralis major and latissimus dorsi muscles,^17^, ^18^ the second between the pectoralis minor and coracobrachialis muscle.^17^, ^18^ During the examination, the patient should be positioned supine with the upper limb abducted and externally rotated, using a pillow to elevate the axilla if needed, in particular for improved access to the posterior region.^17^, ^18^ To assess for joint effusion, a standing position may also be preferred to use gravitational fluid shifts. A broadband linear probe with lower frequencies may be necessary in obese patients or in the presence of significant axillary fat tissue.^17^, ^18^ The entire axillary space from pectoralis major anteriorly to latissimus dorsi posteriorly should be analyzed using a 4‐quadrant model (anteromedial, anterolateral, posteromedial, and posterolateral). Key sonographic landmarks include the neurovascular bundle, with the artery and vein appearing as anechoic rounded structures surrounded by honeycomb‐patterned neural elements of the brachial plexus^17^, ^18^ (Figure 2).

#### Clinical Relevance

Damage to the axillary fascia during mastectomy or lymph node dissection often leads to lymphatic dysfunction and secondary conditions like axillary web syndrome.^19^ US examination provides critical insights into fascial and lymphatic integrity, guiding rehabilitation approaches such as manual lymphatic drainage and fascial treatments.^20^ Maintaining the fascial structure would be essential to prevent complications like shoulder dysfunction or persistent edema (Table 1).

### Subscapular Fascia (Fascia Subscapularis)

#### Anatomy

**Figure 3:**
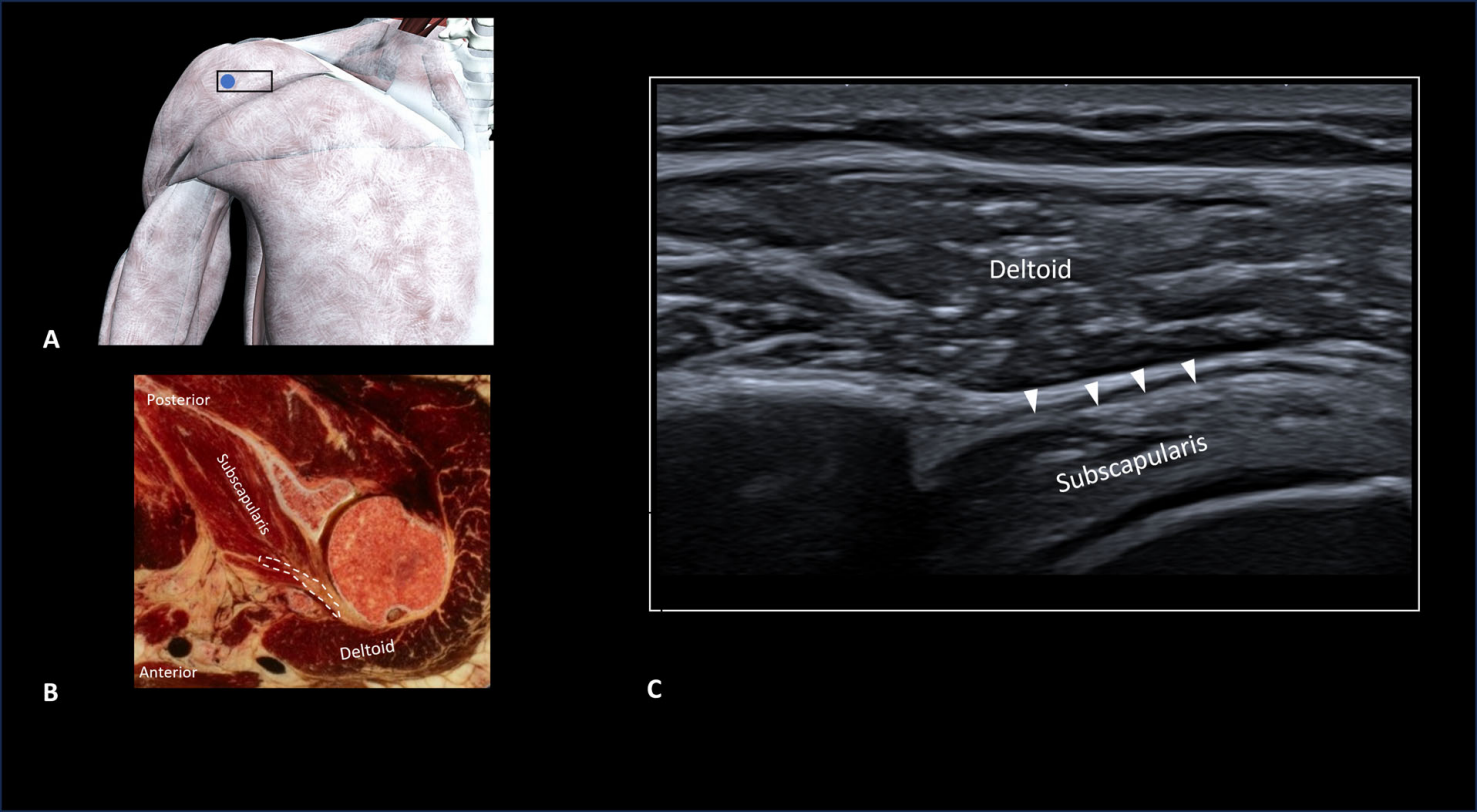
Subscapularis fascia. **A**, Probe positioning. **B**, Coronal anatomical section derived from the Visible Human Project®. *Courtesy of the U.S. National Library of Medicine (NLM), National Institutes of Health* (dotted line: subscpularis fascia). **C**, Ultrasound image revealing the subscapularis fascia (arrowheads).

The subscapularis fascia is a delicate yet distinct aponeurotic layer that envelops the entire subscapular fossa (Figure 3).^8^ Some fibers of the subscapularis muscle originate from its deep surface, integrating the fascial and muscular structures. According to Singer,^21^ the subscapularis fascia is the thinnest among the fascial layers surrounding the scapular musculature; however, despite its thinness, it forms a well‐defined lamina.^8^ Medially, this fascia continues with the rhomboid fascia, while laterally, it maintains continuity with the glenohumeral joint capsule.^8^, ^22^ Functionally, the subscapularis fascia plays a crucial role in the biomechanics of the scapulothoracic joint by providing an optimal gliding plane between the subscapularis and serratus anterior muscles.^8^, ^22^ This interaction facilitates smooth movement and reduces friction during scapular motion. In addition, it forms a robust connection with the subacromial‐subdeltoid and coraco‐acromial bursae, ensuring synchronous movement of the bursae as well. This configuration is essential in mitigating frictional forces that arise from the dynamic coiling of the subscapularis muscle around the coracoid process, in particular of its upper fibers.^22^ During shoulder movements (especially internal rotation and abduction), the subscapularis muscle undergoes significant orientation changes.^8^ The subscapularis fascia along with the subscapularis and subcoracoid bursae, acts as a biomechanical buffer, reducing friction between the superficial muscle fibers and the adjacent structures, including the scapular neck, humeral head, and coracoid process.^8^

#### Ultrasonography

The patient should be positioned supine with the arm externally rotated to optimize visualization of the subscapularis muscle and its fascia. An alternative lateral decubitus position may be used for better access to deeper structures. The scanning should begin at the axillary region, progressing medially along the scapula, using transverse and longitudinal planes to assess the continuity, echogenicity, and thickness. The subscapularis fascia appears as a thin, hyperechoic layer that envelops the subscapularis muscle. It is separated from the fascia of the serratus anterior by an interposed layer of loose connective tissue rich in fat. Key anatomical landmarks include the coracoid process, serving as a reference for scanning the anterior subscapularis fascia and the neurovascular bundle, which has to be evaluated due to its adjacency to the brachial plexus and axillary vessels (Figure 3).

#### Clinical Relevance

Adhesions or fibrosis in the subscapular fascia are common in conditions like frozen shoulder, or scapulothoracic dysfunction.^22^ US can identify restricted movements and areas of thickening, prompting interventions, for example, myofascial hydro‐release and therapeutic stretching. In postoperative patients, monitoring the subscapular fascia can help detect early signs of dysfunction or adhesion formation, improving rehabilitation strategies (Table 1).

### Infraspinatus Fascia

#### Anatomy

**Figure 4:**
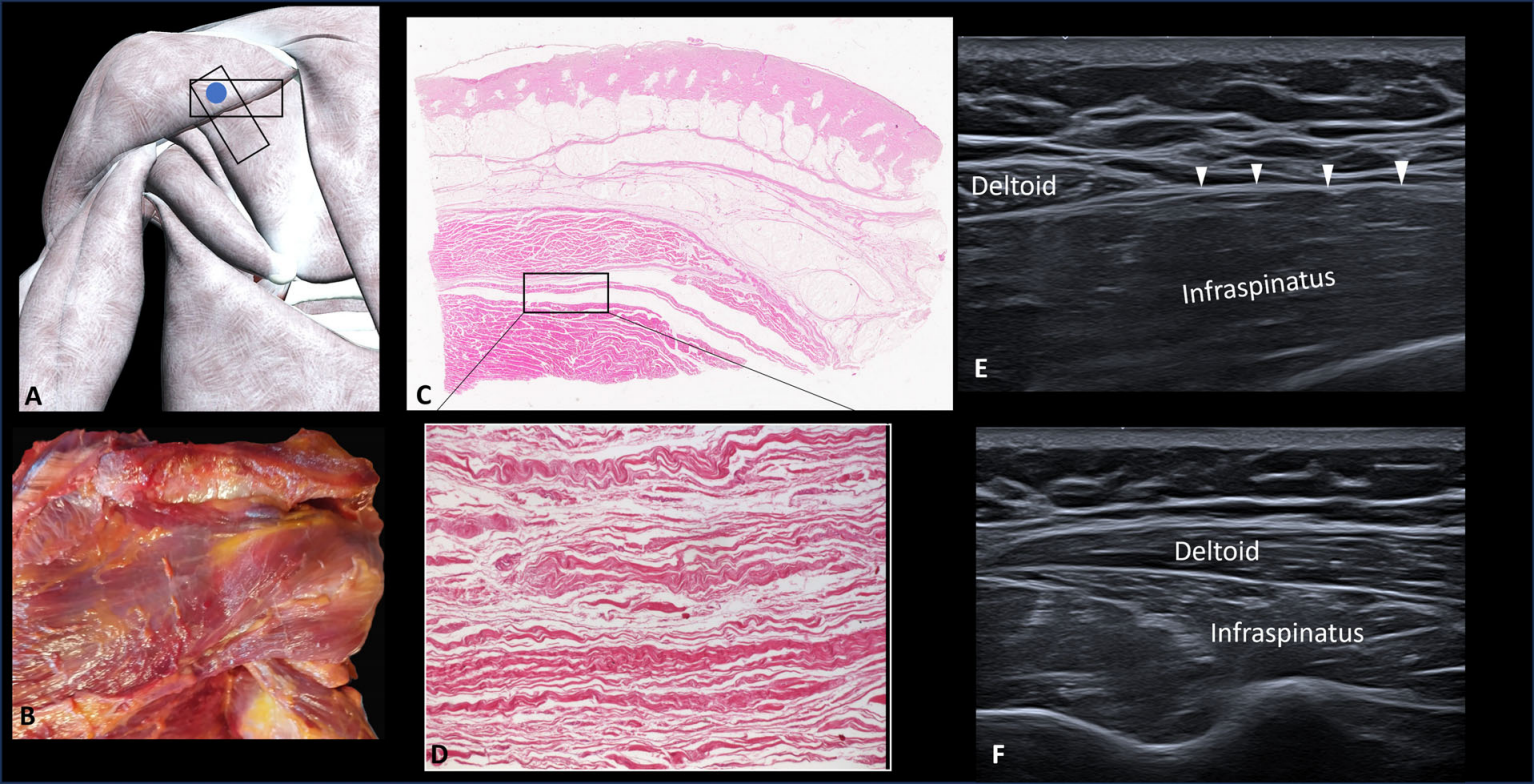
Infraspinatus fascia. **A**, Probe positioning. **B**, Anatomical dissection showing the infraspinatus fascia covering the infraspinatus and teres minor muscles. **C**,**D**, Histological (hematoxylin–eosin staining) and (**D**) high magnification images of the infraspinatus fascia. **E**,**F**, Ultrasound images revealing the infraspinatus fascia (arrowheads).

The infraspinatus fascia is a dense aponeurotic fascia that envelops the infraspinatus and teres minor muscles, anchoring firmly to the perimeter of the infraspinatus fossa (Figure 4).^8^, ^23^ Some fibers of these muscles originate from its deep surface, reinforcing the structural integrity of this fascial layer. While it is partially covered by the deltoid, trapezius and latissimus dorsi muscles; a layer of loose connective tissue ensures independent mobility between these structures, except in specific regions where adhesions create well‐defined lines of force that contribute to shoulder stability and coordinated movements. Notably, Chafik et al^24^ described 2 distinct fascial variants surrounding the teres minor; 1 forming a rigid, localized compartment enclosing both the infraspinatus and teres minor muscles. The primary nerve to teres minor courses through a fascial sling, typically becoming subfascial at an average of 44 mm (range: 25–68 mm) medial to the muscle's insertion. The infraspinatus fascia is important not only for force transmission and myofascial continuity but also because of its involvement in potential fascial nerve entrapments. Abnormalities in the infraspinatus fascia can contribute to shoulder dysfunction and localized muscle atrophy.^25^

#### Ultrasonography

The patient needs to be placed in sitting or prone position, with the arm either resting in neutral or slightly internally rotated position or crossed over the chest to stretch the scapular region. Short‐axis imaging is obtained by placing the probe parallel to the spine of the scapula to visualize the infraspinatus muscle and its overlying fascia. During long‐axis imaging, the probe is aligned with the muscle fibers' direction from medial scapular fossa to its humeral insertion. Dynamic assessment is advisable for the infraspinatus fascia, since shoulder movements such as external rotation or abduction naturally generate gliding between the fascia, the underlying muscle and the scapular surface. Observing these interactions in motion offers valuable insight into how the fascia behaves during real functional activity, revealing subtle restrictions, reduced elasticity, or adhesions that might remain unnoticed in a static image^26^, ^27^ (Figure 4).

#### Clinical Relevance

A particularly stout fascial sling may serve as a site of compression, leading to isolated teres minor atrophy, a condition identified in approximately 3% of patients with shoulder complaints, as reported by Friend et al.^25^ US examination is particularly useful in assessing fascial involvement in shoulder pain syndromes, differentiating myofascial dysfunction from rotator cuff pathology and monitoring post‐surgical or rehabilitative changes in fascial mobility (Table 1).^27^, ^28^, ^29^

### Supraspinatus Fascia

#### Anatomy

**Figure 5:**
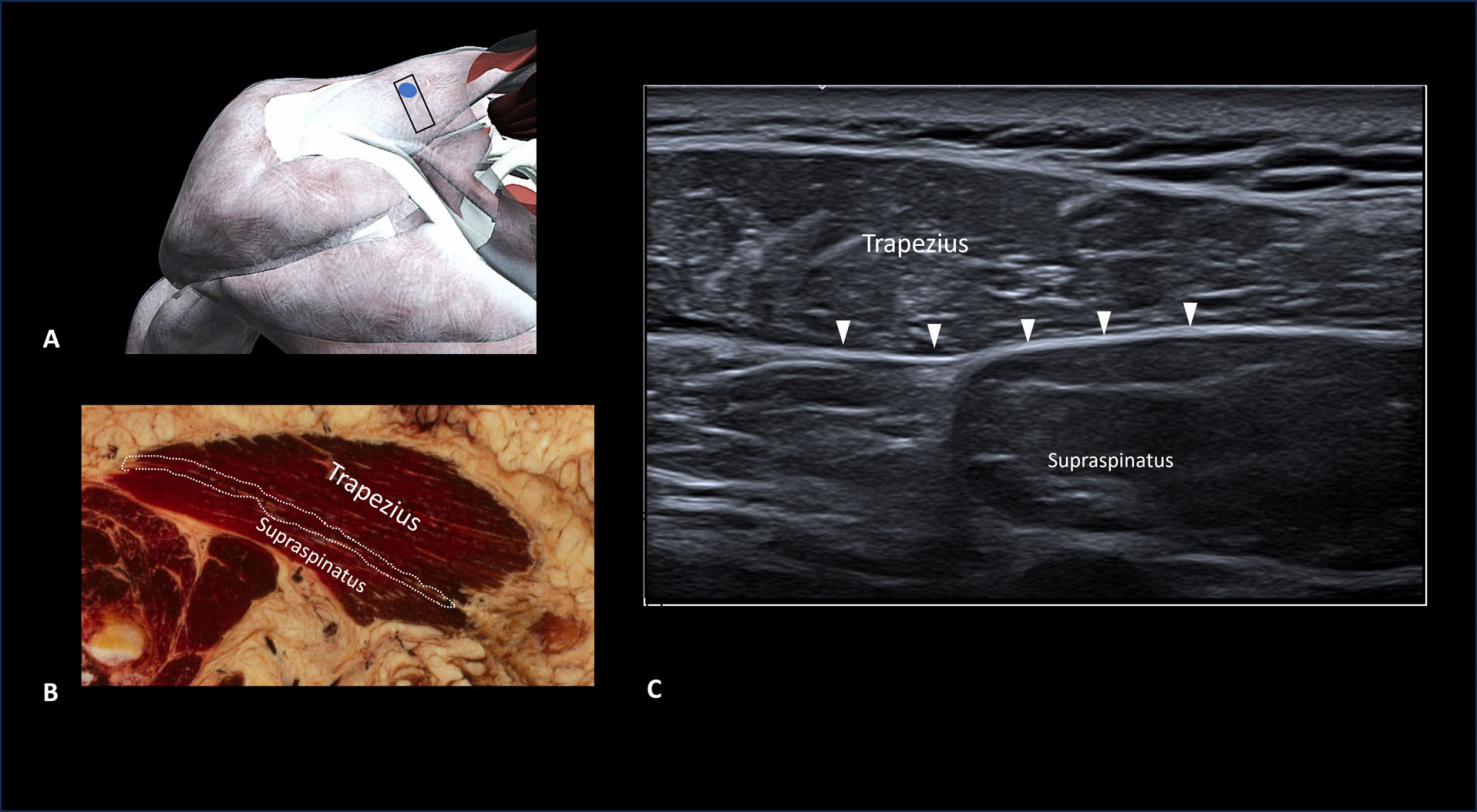
Supraspinatus fascia. **A**, Probe positioning. **B**, Coronal anatomical section derived from the Visible Human Project®. *Courtesy of the U.S. National Library of Medicine (NLM), National Institutes of Health* (dotted line: supraspinatus fascia). **C**, Ultrasound image revealing the supraspinatus fascia (arrowheads).

The supraspinatus fascia is a dense fibrous layer that encloses the osteo‐fibrous compartment containing the supraspinatus muscle (Figure 5).^8^, ^29^, ^30^ Structurally, it provides a site of attachment for muscle fibers on its deep surface and exhibits continuity with adjacent fascial structures, including the levator scapulae fascia in the neck, the clavipectoral fascia anteriorly, and the rhomboid fascia medially.^8^ Over the scapular spine, it partially adheres to and integrates with the infraspinatus fascia, forming a structural continuum that influences force transmission within the shoulder girdle. This fascia demonstrates variable thickness with an average of 0.7 mm, occasionally incorporating adipose tissue as described by Singer.^31^ Duparc et al^29^ identified a thickening at the spinoglenoid notch, which contributes to fascial scapular nerve entrapment, a condition associated with neuropathic shoulder pain and muscular dysfunction. Although the spinoglenoid ligament has traditionally been described as the main site of suprascapular nerve entrapment, Bektas et al^32^ reported its presence in only 15.6% of specimens. This finding challenges the classical view, highlighting that the deep fascia in the spinoglenoid region may equally contribute to fascial nerve compression. Instead, they found that a thickened distal third of the supraspinatus fascia is observed, suggesting that fascial involvement plays a more critical role in suprascapular nerve compression than previously recognized.

#### Ultrasonography

To perform an US scan of the supraspinatus fascia, the patient is positioned seated or in lateral decubitus position, with the arm internally rotated and extended to expose the supraspinatus fossa adequately. In short axis, place the probe parallel to the scapular spine, scanning from medial to lateral, allowing visualization of the supraspinatus muscle, its overlying fascia and adjacent connective tissue. In long axis, align the probe along the muscle fibers, extending from the medial attachment at the supraspinous fossa to the insertion on the greater tubercle of the humerus. This approach highlights the echogenic fascial layer that differentiates from the hypoechoic muscle tissue. Normal supraspinatus fascia appears as a thin, continuous hyperechoic layer over the supraspinatus muscle. Fascial thickening with increased echogenicity and reduced gliding can indicate fibrosis, chronic overuse or strain. About the dynamic US assessment, the patient is instructed to perform passive and active arm movements, such as abduction and external rotation, to assess the gliding and elasticity of the supraspinatus fascia.^26^, ^27^ At the spinoglenoid notch, a thickened fascial band can contribute to suprascapular nerve compression, which can be detected using dynamic sonographic assessments (Figure 5).

#### Clinical Relevance

Comprehensive US examination enables precise evaluation of the supraspinatus fascia, aiding in the diagnosis of myofascial dysfunctions, suprascapular nerve entrapment and shoulder pathology, thus optimizing treatment strategies for clinical and rehabilitation purposes.^29^, ^30^, ^31^ The interplay between fascial thickening and nerve compression should be considered in diagnostic assessments and therapeutic interventions, especially in patients presenting with posterolateral shoulder pain, weakness, or signs of nerve impairment (Table 1).^29^, ^30^, ^31^, ^32^

### Arm—Brachial Fascia

#### Anatomy

The brachial fascia is a dense aponeurotic fascia that envelops the muscles of the upper arm, forming a continuous fibrous sheath comparable to an “evening glove”. It structurally integrates with both proximal and distal fascial planes.^8^, ^33^ This strong fascia contains multidirectional collagen fiber arrangements, which provide both structural reinforcement and dynamic adaptation to muscular forces.^8^, ^33^ Proximally, it integrates with the axillary fascia as well as the fascia of the pectoralis major, deltoid fascia, and latissimus dorsi fascia. Distally, it transitions seamlessly into the antebrachial fascia, ensuring fascial continuity throughout the limb.^8^, ^33^ The brachial fascia is biomechanically significant, as its tension is modulated by myofascial insertions from the shoulder girdle muscles.^34^ Though largely independent in movement from the underlying muscle layers, it forms localized adhesions at key structural points, in particular at the humeral epicondyles.^8^, ^33^, ^35^ Its thickness is different in the various regions (anterior and posterior) and levels; it tends to be thicker posteriorly than anteriorly, aligning with regions subjected to greater mechanical stress.^36^ Structurally, the medial and lateral intermuscular septa originate from the brachial fascia, partitioning the anterior and posterior muscle compartments of the arm.^8^, ^33^, ^35^ The lateral septum serves as a crucial anatomical landmark, as it is traversed by the radial nerve approximately 10 cm proximal to the elbow, marking the transition of a nerve from the posterior to the anterior compartment.^8^ At the elbow, the brachial fascia is reinforced by anterior and posterior retinacula, which represent structural thickening formed by specific myofascial expansions.^33^ The anterior retinaculum consists of the lacertus fibrosus, a fibrous lamina arising from the distal biceps tendon. The lacertus fibrosus extends into the antebrachial fascia, forming oblique and longitudinal reinforcements.^8^, ^33^ The posterior elbow retinaculum is reinforced by expansions from triceps brachii, which integrate into the antebrachial fascia. These myofascial expansions, described by Windisch et al^37^ and Keener et al^38^, originate from the lateral head of the triceps. Posteriorly, the brachial fascia extends toward the border of the ulna and blends with the anconeus fascia, providing a firm anchorage for the triceps tendon and contributing to the stabilization of the posterolateral aspect of the elbow.

#### Ultrasonography

**Figure 6:**
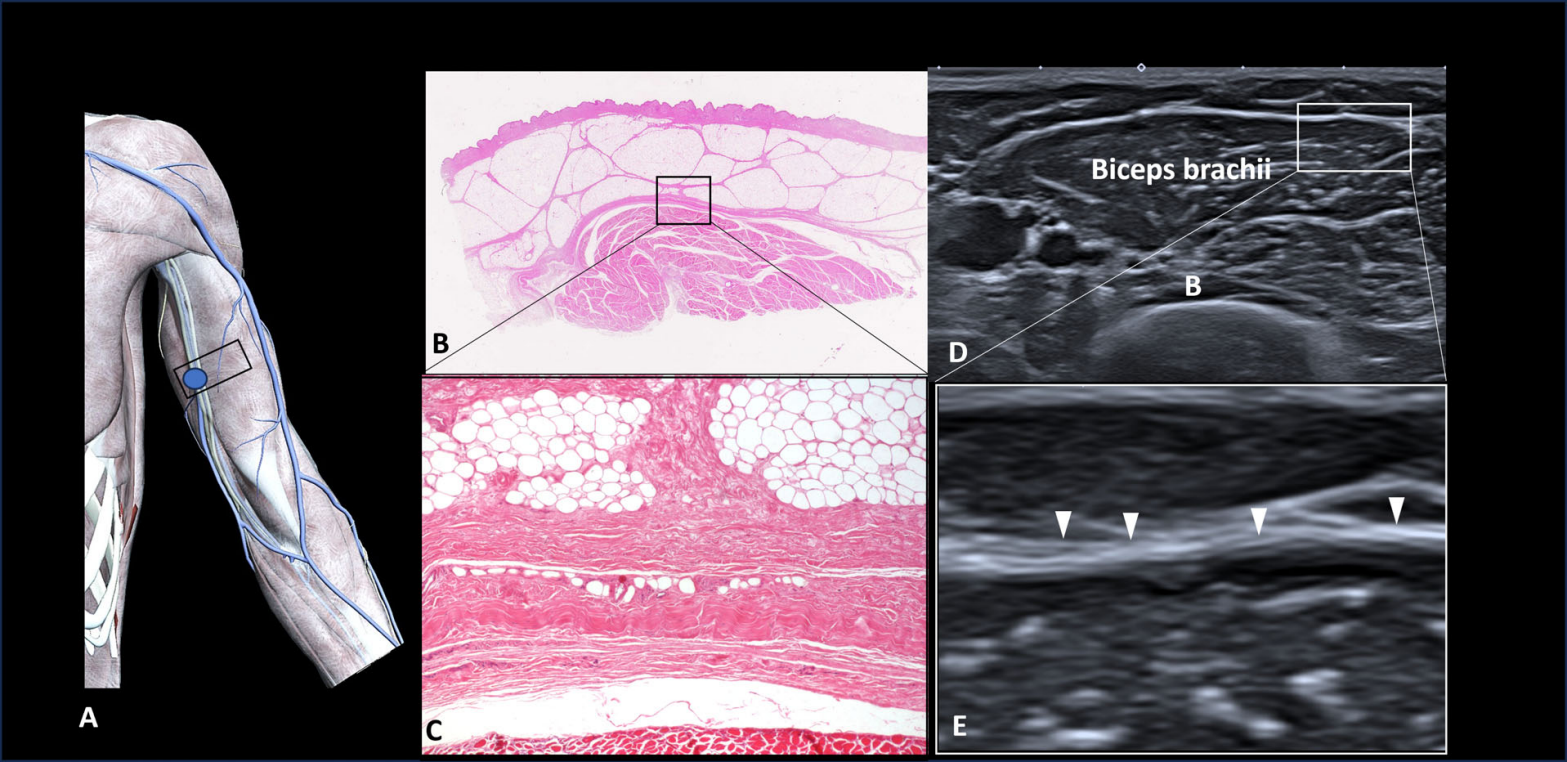
Brachial fascia. **A**, Probe positioning. **B**,**C**, Histological (hematoxylin–eosin staining) and (**C**) high magnification images of the brachial fascia. **D**,**E**, Ultrasound images revealing the brachial fascia (arrowheads). B, brachialis muscle.

The US examination of the brachial fascia requires a standardized approach to ensure accurate visualization and measurement of its structure and thickness.^36^ For the anterior brachial fascia, the patient is in supine position with the upper limb in neutral position, while for the posterior side the patient is in prone position with the arm relaxed in a neutral posture.^36^ For the proximal anterior level, position the probe transversely proximal to the anterior arm, identifying the brachial artery and median nerve between the brachialis and biceps brachii muscles.^33^ The brachial fascia appears as a hyperechoic linear band superficial to the biceps brachii and brachialis muscle. For the distal level, move the probe, with a maneuver of lifting, along the arm, maintaining the brachialis muscle as a reference.^36^ It is crucial to observe the integration of fascia with intermuscular septa. For the posterior region, position the probe transversely over the posterior upper arm, identifying the triceps brachii muscle bellies. The radial nerve can serve as a key landmark in the spiral groove.^36^ Moving distally, the fascial sublayers of the deep fascia merging with the intermuscular septa can be tracked. For dynamic assessment, the patient is asked to actively flex and extend the elbow to observe fascial gliding. According to Pirri et al^36^ the normal brachial fascia thickness is 0.81 ± 0.2 mm in the posterior region, significantly thicker than the anterior region, 0.61 ± 0.11 mm; while fascial thickening or hyperechogenicity suggests fibrosis, chronic strain, chronic overuse, scarring, or densification (Figure 6).

#### Clinical Relevance

Clinically, the brachial fascia is integral to force transmission and proprioception within the upper limb. Studies by Van der Wal^39^ emphasize that deep muscular fasciae and muscular structures function as a unified biomechanical system, rather than isolated elements also in the upper limb. The brachial fascia's involvement in radial nerve entrapment syndromes is also significant, as fascial densifications at the lateral intermuscular septum or the arcade of Struthers may contribute to nerve compression and mobility restrictions, particularly in cases of humeral fractures that remodel the fascial layers or excessive fascial tension. By following this standardized US approach, physiatrists and clinicians can reliably monitor fascial changes post‐surgery or trauma and finally evaluation of compartment syndrome risks (Table 2).^40^, ^41^, ^42^, ^43^, ^44^

**Table 2:** Deep Fasciae of the Brachial and Antebrachial Regions: Integrated Anatomical, Sonographic, and Clinical Features

Type of Deep Fascia	Average/Range Thickness	Sonographic Landmarks	Ultrasound Appearance	Clinical/Functional Relevance
Brachial fascia	0.81 ± 0.20 mm (posterior)—0.61 ± 0.11 mm (anterior)	Proximal anterior arm: between biceps brachii and brachialis, near brachial artery and median nerve Posterior arm: over triceps bellies, radial nerve in spiral groove Fascial continuity with axillary fascia proximally and antebrachial fascia distally	Hyperechoic linear band superficial to biceps and triceps; merges with intermuscular septa; posterior layer thicker and more echogenic. Dynamic flexion–extension shows gliding	Transmits tensile forces and proprioceptive input within upper limb. Fascial densification or adhesions may cause pain, limited range, or nerve entrapment (radial nerve at lateral septum, arcade of Struthers). US enables detection of fibrosis, chronic overuse, post‐traumatic scarring, and compartment‐syndrome monitoring
Antebrachial (forearm) fascia	0.70 ± 0.20 mm (anterior)—0.71 ± 0.20 mm (posterior)	Anterior proximal scan: pronator teres, FCR, FDS; median nerve between pronator heads Distal anterior: FDP, palmaris longus, FCU; transition into flexor retinaculum Posterior: ECRL/ECRB/ED/ECU and radial nerve branches	Multilayered hyperechoic bands enveloping superficial and deep muscle groups; continuous with wrist retinacula and dorsal hand fascia; homogeneous thickness across regions	Fundamental for load transmission and compartmental integrity. Altered fascial gliding or thickening correlates with myofascial pain, chronic strain, or entrapment syndromes. US assists in differentiating fascia‐related pathologies, guiding hydrodissection and monitoring post‐injury remodeling. Highly vascular fascia useful in reconstructive flap planning

### Forearm—Antebrachial Fascia

#### Anatomy

The antebrachial fascia is a dense, multi‐sublayered fascia structure enveloping the forearm muscles, forming a fibrous cuff that integrates with both deep and superficial muscular layers.^8^ It acts as a compartmental separator, dividing the forearm into anterior, lateral, and posterior compartments, with distinct septa separating the deep from the superficial muscle layers.^8^ The antebrachial fascia is formed by collagen fiber bundles oriented in multiple directions, providing both stability and adaptability to mechanical stress. In the proximal forearm, several muscles insert into its deep surface, contributing to force transmission and fascial tension.^8^ Conversely, in the distal forearm, it is easily separable from underlying muscle but remains firmly anchored to the radial and ulnar styloid processes, reinforcing structural integrity. Functionally, the antebrachial fascia transitions into the dorsal fascia of the hand, contributing to thenar and hypothenar fasciae, as well as forming the deep layer of the contributing to thenar and hypothenar fasciae, as well as forming the deep layer of the palmar aponeurosis. A notable anatomical feature is the perforation by the palmaris longus tendon, which traverse the fascia before inserting into the palmar aponeurosis. The wrist region presents additional structural reinforcements, through the flexor and extensor retinacula, which stabilize tendons during motion. These fibrous bands, consist of cross‐layered fibrous bundles oriented in mediolateral and lateromedial directions. These retinacula are highly innervated, playing a pivotal role in proprioception and motor coordination. A critical distinction must be made between the flexor retinaculum and the transverse carpal ligament.^45^ The flexor retinaculum serves as a reinforcement of the antebrachial fascia, whereas the transverse carpal ligament is an independent fibrous‐osseus structure, spanning between the hamate and pisiform medially and the scaphoid and trapezium laterally.^45^

#### Ultrasonography

**Figure 7:**
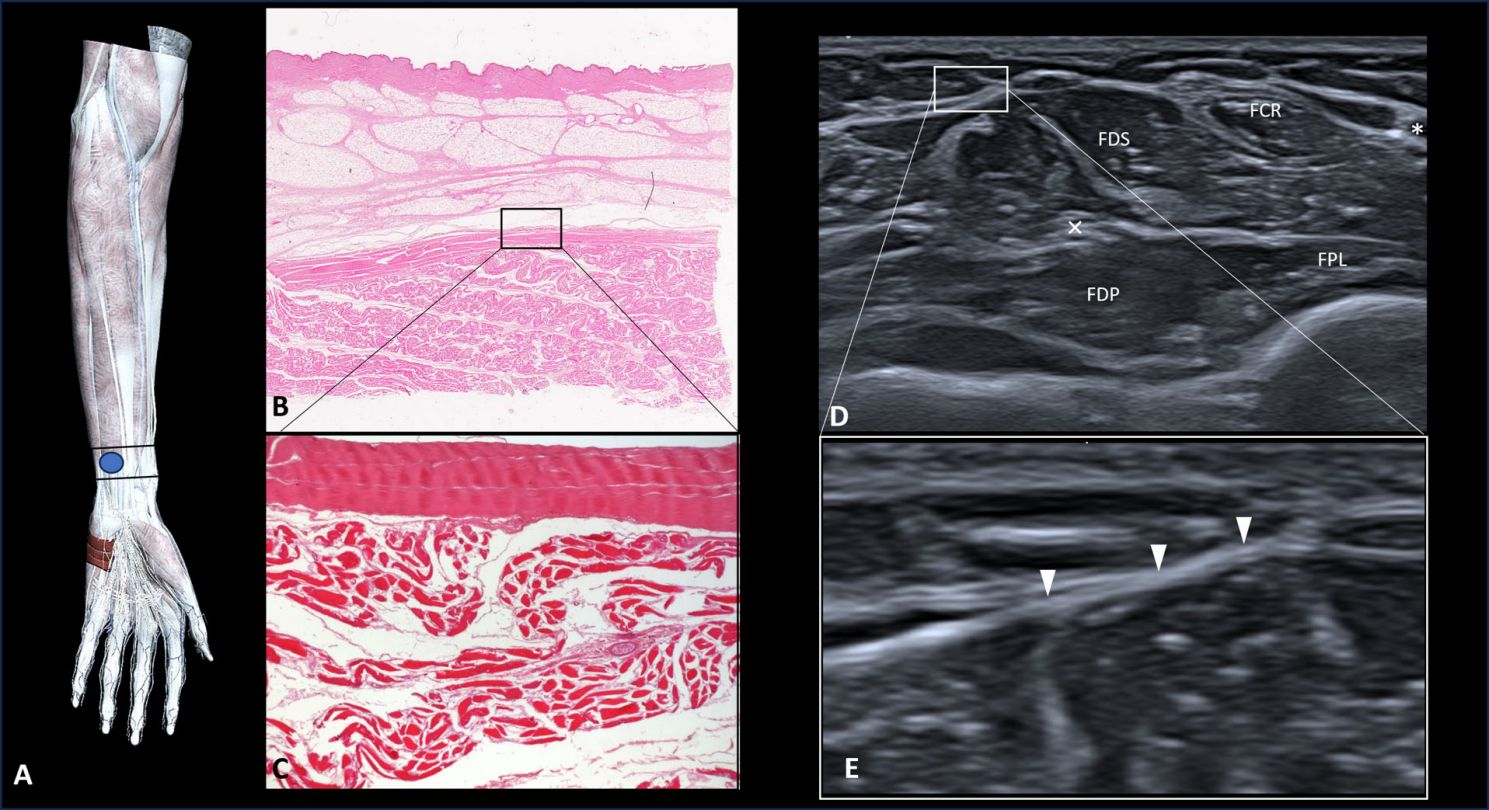
Antebrachial fascia. **A**, Probe positioning. **B**,**C**, Histological (Hematoxylin–eosin staining) and (**C**) high magnification images of the antebrachial fascia. **D**,**E**, Ultrasound images revealing the antebrachial fascia (arrowheads). FDS, flexor digitorum superficialis muscle; FDP, flexor digitorum profundus muscle; FPL, flexor pollicis longus muscle; FCR, flexor carpi radialis muscle; ×, median nerve; *, radial artery.

To perform an US examination of the antebrachial fascia, a structured protocol and optimal probe placement are essential for accurate imaging and fascial assessment.^36^ For the anterior antebrachial fascia scan, the position of the patient is supine with the forearm supinated. Ensuring the limb is in a fully relaxed position is needed to reduce muscle‐induced fascial tension.^36^ The average thickness of the antebrachial fascia is 0.7 ± 0.2 mm for the anterior region and 0.71 ± 0.2 mm for the posterior region, indicating at this level a relatively uniform distribution of fascial thickness throughout the forearm. For the anterior region, in the proximal scan, the probe is placed axially over the proximal volar forearm, identifying the pronator teres, flexor carpi radialis (FCR), and flexor digitorum superficialis (FDS). The median nerve can be seen passing between the 2 heads of the pronator teres. The fascial layers between the superficial and deep forearm muscles are assessed. In the distal scan, the probe is moved distally where the flexor digitorum profundus (FDP), palmaris longus, and flexor carpi ulnaris (FCU) become more prominent.^36^ The antebrachial fascia's transition into the flexor retinaculum is identified. For the posterior region, in the proximal scan, the probe is positioned transversely over the dorsal forearm, visualizing the extensor digitorum (ED), extensor carpi radialis brevis (ECRB), and extensor carpi radialis longus (ECRL). The radial nerve and its branches as landmarks are also located. In the distal scan, move the probe distally, capturing the extensor carpi ulnaris (ECU), ED, and abductor pollicis longus (APL). Identify the fibrous expansion of ECU into hypothenar fascia, as well as its continuity with the dorsal carpal fascia.^36^ The patient is instructed to actively flex and extend the wrist and fingers, assessing fascial gliding. Loss of gliding suggests fascial adhesions, nerve entrapments, or compartment syndromes (Figure 7).

#### Clinical Relevance

Clinically, the antebrachial fascia is integral to upper limb biomechanics, serving as a force‐transmitting structure that integrates muscular contractions into coordinated hand and wrist movements. Its involvement in pathological conditions, such as compartment syndromes,^46^ fascial densification, and myofascial pain syndromes, underscores the importance of accurate US examination. Targeted therapeutic interventions, including hydrodissection or hydrorelease, are essential to optimize functional outcomes. Moreover, this fascia plays a crucial vascular role, serving as a conduit for fasciocutaneous flap perfusion. Studies by Tao et al^47^ demonstrate that the vascular network within the deep forearm fascia is significantly denser than that of the superficial fascia, highlighting its importance in surgical reconstructive application (Table 2).

## Hand

### Palmar Fascial Complex

#### Anatomy

The palmar fascial complex is an intricate system that integrates 5 primary components: the palmar aponeurosis, deep palmar fascia, adductor fascia, thenar and hypothenar fasciae, and the septa interconnecting these elements.^8^ The palmar aponeurosis is a specialized aponeurotic fascia, composed of 2 distinct sublayers discernible through histological examination.^48^ The superficial sublayer consists exclusively of longitudinally arranged collagen fibers, while the deep layer contains transversely oriented collagen fibers, which reinforce the structure distally and contribute to the formation of the deep transverse metacarpal ligament.^8^ This palmar aponeurosis is tightly adhered to the skin via dense retinacula cutis, which facilitates force transmission from the skin to the deep fascial structures and underlying bones. The system is activated upon palmaris longus muscle contraction or metacarpophalangeal joint extension, enabling dynamic control of palmar skin mobility. A fundamental feature of the palmar fascial complex is its septal system, which includes: (1) 2 longitudinal marginal septa connecting the palmar aponeurosis to the deep palmar fascia; (2) 7 vertical septae, first described by Legueu and Juvara (1892),^49^ segmenting the central compartment of the hand into 8 functional units. These septa create specialized compartments: 4 accommodating the flexor tendons of digits 2–5 and 4 containing the lumbrical muscles with their corresponding neurovascular structures.

The deep palmar fascia, also referred to as the interosseous fascia, is a reinforcing structure located beneath the lumbrical muscles and over the interosseous compartments. Proximally, it integrates with the capsular ligaments of carpal joints, while distally, it merges into the fibrous sheaths of the flexor tendons, ensuring stability and controlled tendon gliding.^8^

The thenar and hypothenar fasciae are classified as epimysial fasciae, firmly adhering to their underlying muscles. Unlike the palmar aponeurosis, these fasciae lack a distinct laminar structure, as observed by Ling and Kumar.^50^ Instead, they contain loose connective tissue layers interspersed between muscle fibers, allowing intrinsic hand muscles gliding and flexibility. These fasciae continue proximally into the flexor retinaculum, contributing to structural reinforcement and modulating fascial tension through their myofascial expansions. A notable anatomical feature is the fibrous expansion from the abductor digiti minimi to the dorsal fascia of the fifth digit. This structure transmits muscular tension to the dorsal metacarpophalangeal and interphalangeal joints, enhancing digit stabilization and coordinated hand motion.

The adductor pollicis fascia is a thin but functionally significant fascial layer, extending radially from the third metacarpal to the first metacarpal. It serves as a reinforcement adjacent to the flexor pollicis longus tendon, integrating distally with the fascia covering the first dorsal interosseous muscle. Additionally, selected adductor pollicis fibers originate directly from this fascia, emphasizing its role in force transmission and thumb stabilization.

#### Ultrasonography

To perform an US examination of the palmar fascial complex the patient has to be seated with the forearm supinated, hand relaxed and fingers slightly abducted to expose the palmar surface. For deeper palmar fascia and adductor fascia, mild wrist extension optimizes US imaging by reducing flexor tendon overlap. A high‐frequency hockey stick linear probe ensures optimal fascial detail. A US imaging depth between 10 and 25 mm is recommended, with adjustments made occurring to the target structure to optimize image resolution and acoustic focus. Gain should be optimized to differentiate hyperechoic fascia from hypoechoic muscles.

##### Palmar Aponeurosis

**Figure 8:**
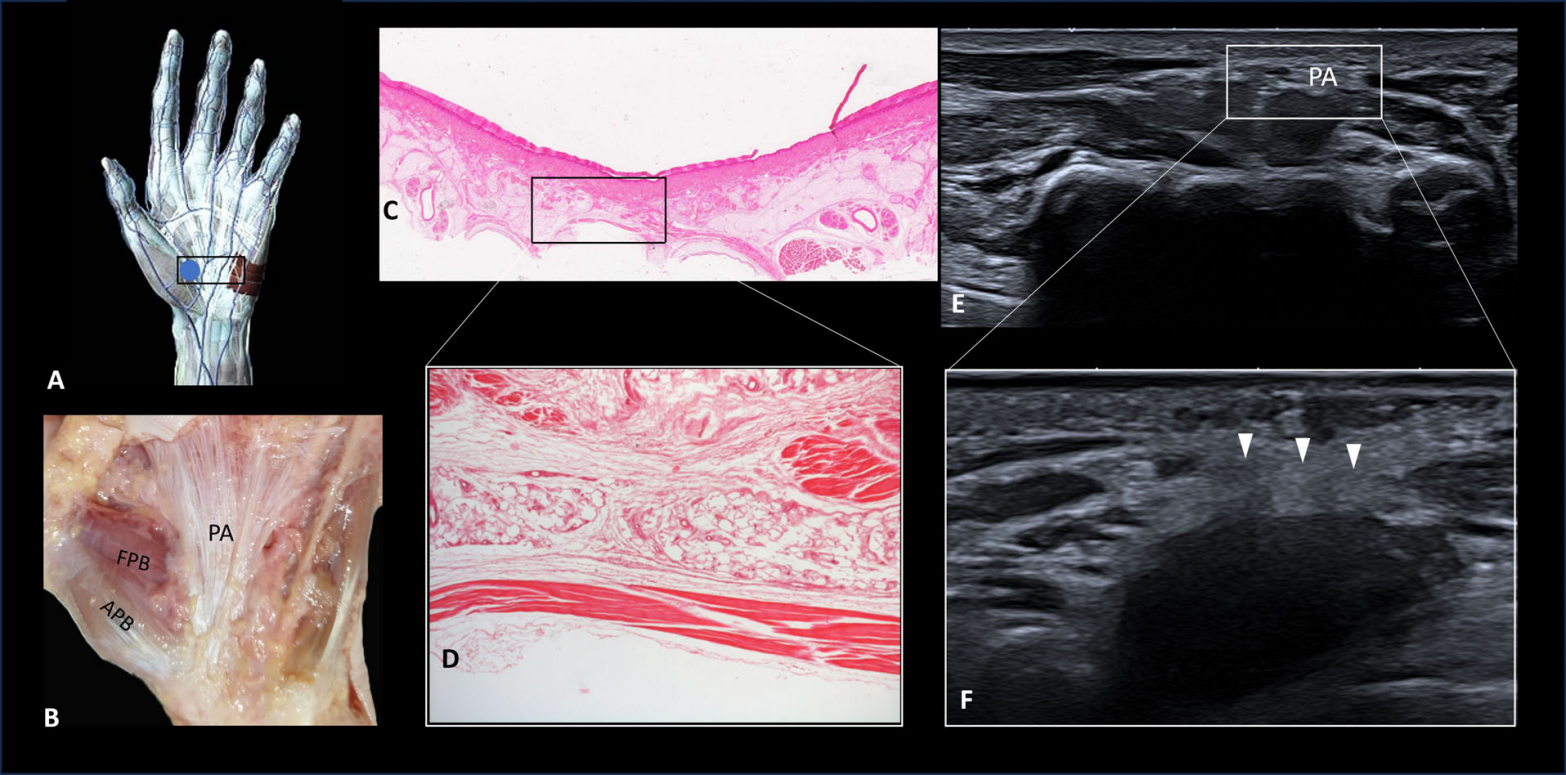
Palmar aponeurosis. **A**, Probe positioning. **B**, Anatomical dissection showing the palmar aponeurosis as a dense fibrous layer. **C**,**D**, Histological (hematoxylin–eosin staining) and (**D**) high magnification images of the palmar aponeurosis. **E**,**F**, Ultrasound image (*transverse view*) displaying the palmar aponeurosis as a hyperechoic structure (arrowheads). PA, palmar aponeurosis; FPB, flexor pollicis brevis muscle; APB, abductor pollicis brevis.

The probe is placed in short‐axis over the palmar surface just proximal to the metacarpophalangeal (MCP) joints to see the different structures. It is then rotated 90°, in long axis, to identify the 2 distinct sublayers: (1) superficial: hyperechoic, longitudinally oriented collagen fibers; (2) deep: contains transverse collagen fibers, forming the deep transverse metacarpal ligament that is appreciated with a new rotation in short axis. Normal palmar aponeurosis appears as a thin, hyperechoic linear band, while in pathological changes it appears thickened (Dupuytren's contracture) with loss of gliding (Figure 8).

##### Deep Palmar Fascia

**Figure 9:**
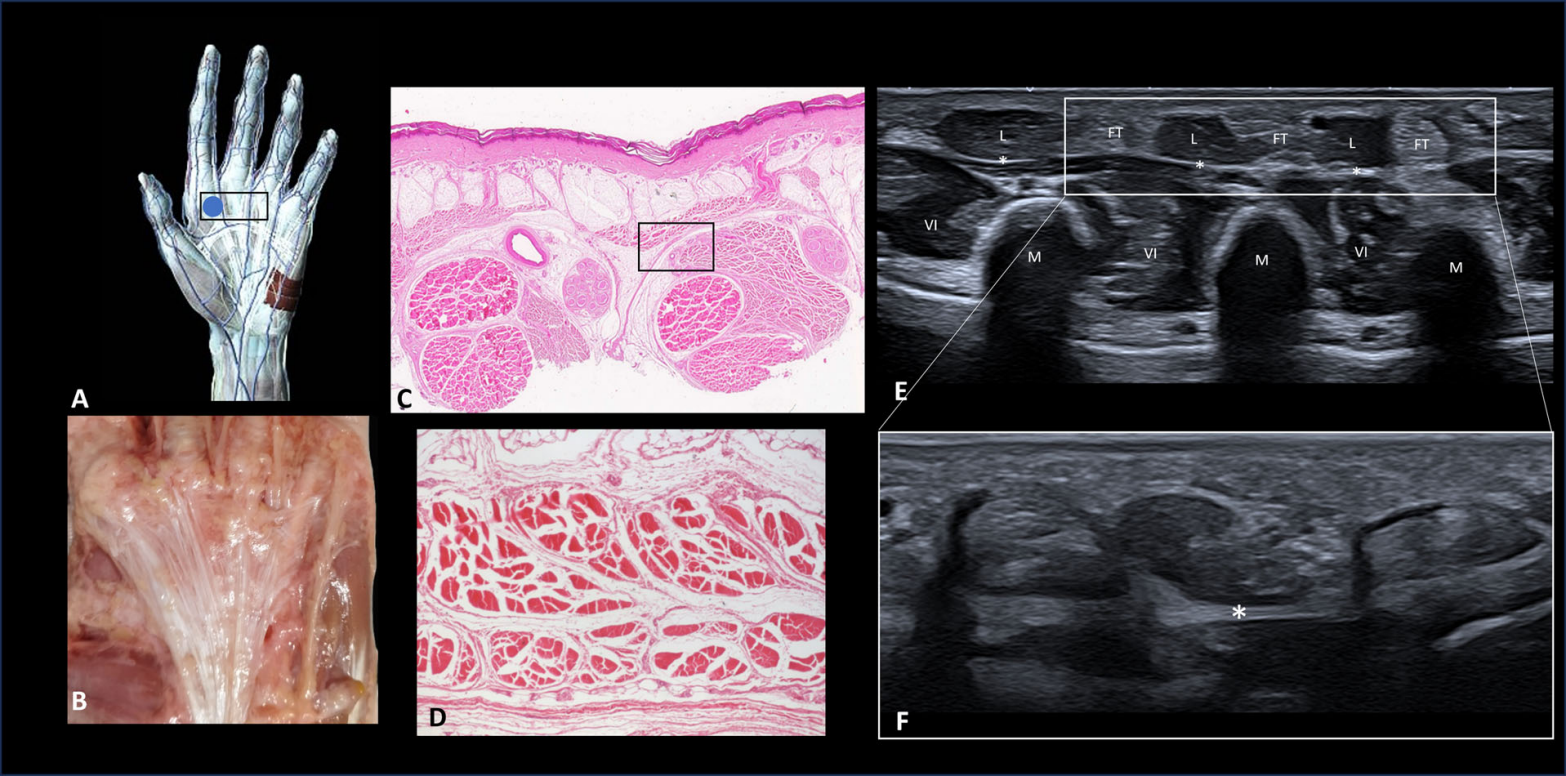
Deep palmar fascia. **A**, Probe positioning. **B**, Anatomical dissection showing the deep palmar fascia as a dense fibrous layer below the lumbrical and flexor tendons and above the interosseous muscles. **C**,**D**, Histological (Hematoxylin–eosin staining) and (**D**) high magnification images of the palmar aponeurosis. **E**,**F**, Ultrasound image (*transverse view*) displaying the deep palmar fascia as a hyperechoic structure. L, lumbrical muscles; FT, flexor tendons; M, metacarpal bones; VI, ventral interossei muscles.

The probe is placed transversely (short‐axis) over mid‐palmar region. The deep palmar fascia is seen as a hyperechoic band, situated below the lumbrical and flexor tendons and above the interosseous muscle. Fascial continuity with the metacarpal bones and the palmar aponeurosis is assessed. Normally, it appears as hyperechoic layer above the interossei muscles, while in pathological conditions it appears fibrotic with compartmental adhesions (Figure 9).

##### Thenar and Hypothenar Fasciae

**Figure 10:**
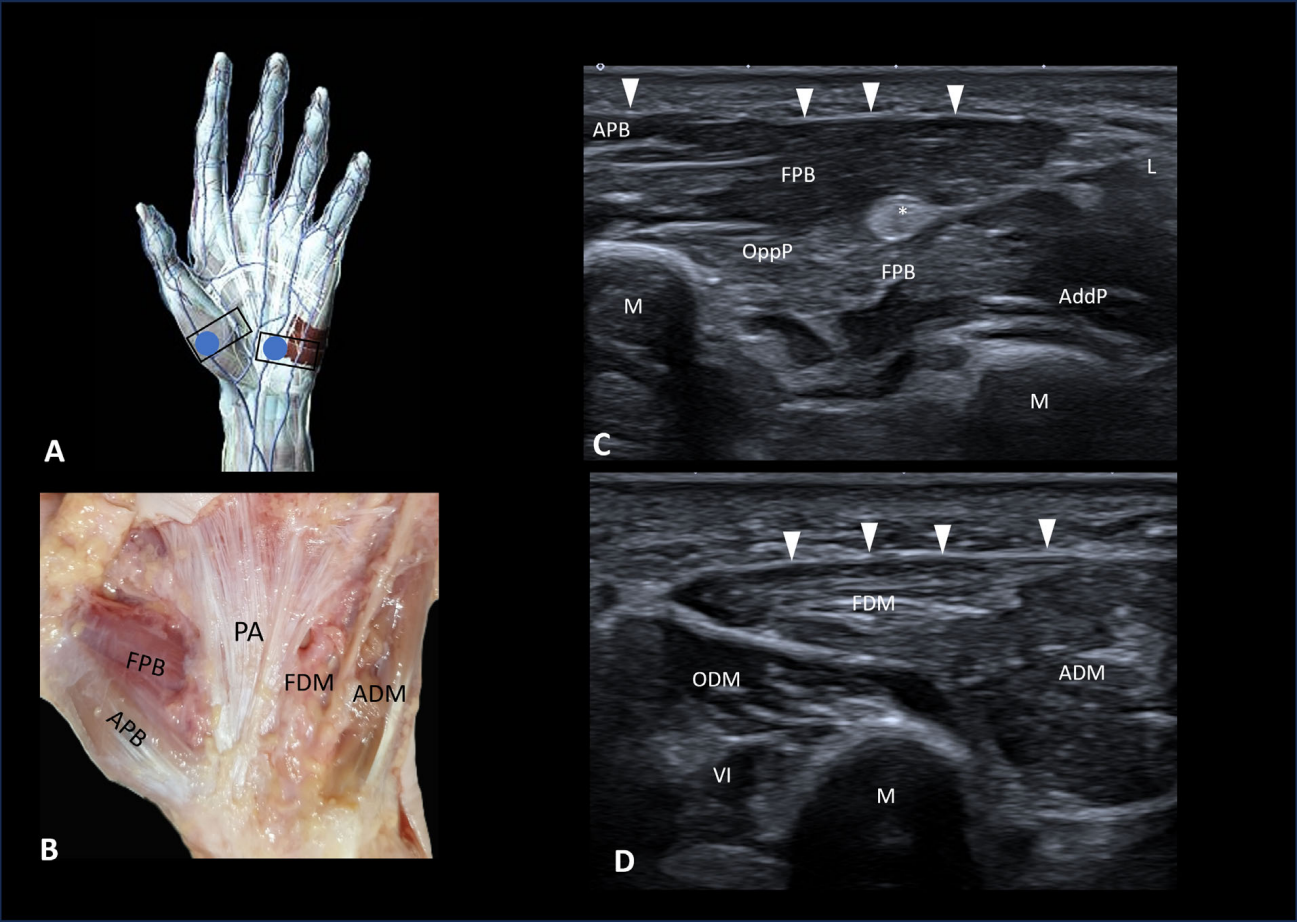
Thenar and hypothenar fasciae. **A**, Probe positioning. **B**, Anatomical dissection showing the thenar and hypothenar fasciae, appearing as epimysial layers, often less distinct from the underlying muscles. **C**,**D**, Ultrasound images revealing the thenar and hypothenar fasciae (arrowheads) as a thin hyperechoic band. APB, abductor pollicis brevis muscle; FPB, flexor pollicis brevis muscle; OppP, opponens pollicis muscle; L, lumbricalis muscle; AddP, adductor pollicis muscle; *, flexor pollicis longus tendon; FDM, flexor digiti minimi muscle; ADM, abductor digiti minimi muscle; VI, ventral interosseus muscle; M, metacarpal bone.

While scanning the thenar fascia, the probe is positioned at the base of the thumb, in short and long axes. For the hypothenar fascia, the probe is placed over the medial palm, tracking continuity with the flexor retinaculum. These fasciae appear as epimysial layers, often less distinct from underlying muscles. Normally they appear less distinct with epimysial continuity, while they are altered in fasciitis and myofascial densification (Figure 10).

### Adductor Fascia

It is scanned radially between the first and third metacarpals, near the ulnar side of the flexor pollicis longus tendon. The fascial extension from the third to the first metacarpal is identified. Normally, it appears as a hyperechoic layer near the first metacarpal bone, while it approaches myofascial entrapments and fascial tears.

For the dynamic assessment, the patient has to perform flexion and extension of the fingers to evaluate palmar aponeurosis tensioning and fascial gliding. Sono‐palpation, color/power Doppler, and sono‐elastography are useful to differentiate normal fascia from densified or fibrotic areas, particularly in Dupuytren's contracture progression.

#### Clinical Relevance

The palmar fascial complex is fundamental to hand biomechanics, proprioception and mechanical load distribution. It ensures efficient force transmission between muscular contractions and the skeletal framework, aids in coordinated finger movements and plays a vital role in maintaining palmar skin stability during grasping and fine motor control. Dysfunction of these fascial structures, including fibrotic thickening (as Dupuytren's disease) can result in restricted hand function and deformities, highlighting the importance of a detailed anatomical understanding for both surgical interventions and rehabilitative strategies.^51^ Moreover, the fascia's compartmentalization of lumbrical muscles and flexor tendons facilitates precise control of digital flexion and grip dynamics.^50^ US examination of these fasciae provides a real‐time, high‐resolution imaging modality to assess fascial pathologies, distinguishing between nerve entrapments—such as carpal tunnel and Guyon's canal syndromes—fascial compression; this differentiation is crucial for optimizing treatment strategies (Table 3).^52^

**Table 3:** Deep Fasciae of the Hand Region: Integrated Anatomical, Sonographic, and Clinical Features

Type of Deep Fascia	Average/Range Thickness	Sonographic Landmarks	Ultrasound Appearance	Clinical/Functional Relevance
Palmar aponeurosis	0.6–1.0 mm (variable by site; may reach >2 mm in Dupuytren's disease)	Central palm, proximal to MCP joints; overlying flexor tendons and lumbricals; continuity with palmaris longus tendon and flexor retinaculum	Two sublayers visible: superficial (longitudinal, hyperechoic) and deep (transverse, forming deep transverse metacarpal ligament); smooth, thin band in normal state	Thickening and loss of gliding indicate fibrosis or Dupuytren's contracture; US guides fascial release, hydrodissection, and monitoring of disease progression
Deep palmar fascia (interosseous fascia)	0.3–0.6 mm	Mid‐palmar region, between lumbricals/flexor tendons and interossei; continuity with metacarpal periosteum	Hyperechoic layer deep to lumbricals and flexor tendons, superficial to interossei; merges with palmar aponeurosis and tendon sheaths	Fibrotic changes or adhesions restrict tendon gliding, contributing to compartmental pain or stiffness; US helps visualize deep adhesions and guide percutaneous release
Thenar and hypothenar fasciae	Variable; thin epimysial fasciae (0.3–0.5 mm)	Thenar: base of thumb; Hypothenar: medial palm; both continuous with flexor retinaculum	Less distinct hyperechoic epimysial lines enveloping intrinsic muscles; loose connective tissue may cause acoustic heterogeneity	Densification or fasciitis may alter muscle gliding and contribute to pain or weakness; US detects inflammatory or fibrotic changes, guiding manual or percutaneous therapy
Adductor pollicis fascia	<0.5 mm	Between 1st and 3rd metacarpals, radial to flexor pollicis longus tendon	Hyperechoic linear layer along radial side of first metacarpal; continuity with first dorsal interosseous fascia	Ensures thumb stabilization and transmits tension from adductor pollicis; fascial tears or fibrosis may limit thumb adduction and precision grip
Dorsal fascia—superficial lamina	0.3–0.6 mm	Over extensor tendons, proximal continuity with extensor retinaculum, distal with dorsal digital fascia	Hyperechoic line covering extensor tendons; separated by thin hypoechoic layer of loose connective tissue from deep lamina	Thickening or adhesions restrict tendon excursion, causing dorsal hand stiffness and extensor tendinopathy; US crucial for pre/post‐surgical evaluation
Dorsal fascia—deep lamina	0.4–0.7 mm	Above dorsal interossei, adherent to metacarpal periosteum	Hyperechoic band immediately above bone; merges with interosseous fascia	Fibrosis or post‐traumatic scarring compromises extensor glide and proprioception; US detects fascial entrapment, inflammation, and altered sliding mechanics
Deep fasciae of the fingers	Very thin, multilayered fibrous lamina (<0.5 mm on average); locally thickened at joint retinacula	Over phalanges and joints; volar and dorsal aspects; surrounding flexor and extensor tendons; reinforced at MCP, PIP, and DIP levels by sagittal and oblique retinacula	Hyperechoic continuous sheath enveloping tendons; thickened, well‐defined hyperechoic bands at joint levels corresponding to retinacular thickenings; minimal interposed subcutaneous fat	Ensures mechanical precision of digital motion by coupling skin and tendon movement; lack of superficial fascia prevents shear displacement. Fibrosis or densification limits tendon glide and digital extension; US identifies adhesions, fascial thickening, or retinacular dysfunction
Extensor retinacular system (transverse–sagittal–oblique fibers)	Not numerically specified; thickened bands up to 0.6–0.8 mm in some dorsal joint areas (based on comparative US reports)	Over MCP joint dorsum; sagittal bands lateral to extensor digitorum tendon; oblique fibers forming triangular lamina distal to sagittal band	Linear hyperechoic structures overlying extensor tendons; oblique fibers seen crossing dorsally; dynamic flexion‐extension demonstrates tendon containment within retinacular sling	Stabilizes extensor tendon excursion and coordinates phalangeal extension; fascial injury or adhesion can mimic “triggering” or post‐traumatic stiffness; US assesses tendon tracking and detects partial tears or fibrosis
Volar retinacular and oblique ligament system	<0.5 mm	Along volar aspect of phalanges, adjacent to flexor sheath; connects to interosseus and lumbrical insertions	Hyperechoic bands parallel to flexor tendons, blending with pulley system (A2–A4)	Coordinates distal phalangeal motion; involvement in trigger finger and post‐surgical adhesions; US identifies fascial‐ligamentous entrapments and loss of gliding

### Deep Fasciae of the Dorsal Region

#### Anatomy

The dorsal fascia of the hand consists of 2 distinct fascial sublayers, playing a fundamental role in the structural integrity and coordination of the extensor tendons apparatus. The superficial lamina envelops the extensor tendons, ensuring continuity proximally with the extensor retinaculum and distally with the dorsal digital fascia.^8^ Meanwhile, the deep lamina is positioned above the dorsal interosseous muscles, where it fuses with the periosteum of the metacarpal bones, providing structural reinforcement to the dorsal hand compartment.^8^, ^53^ Despite their thin thickness, both sublayers of the dorsal fascia exhibit aponeurotic characteristics, facilitating load transmission and extensor tendon stabilization. The presence of loose connective tissue between these layers is crucial for allowing smooth tendon gliding, minimizing mechanical friction during finger and wrist extension. Additionally, strong adhesions between the superficial lamina and the intertendinous connections create a functional interplay between fascia and extensor tendons, optimizing force distribution and coordinated motion.^54^ Furthermore, myofascial expansions from extensor carpi radialis longus into the deep lamina reinforce the dorsal fascia.

#### Ultrasonography

**Figure 11:**
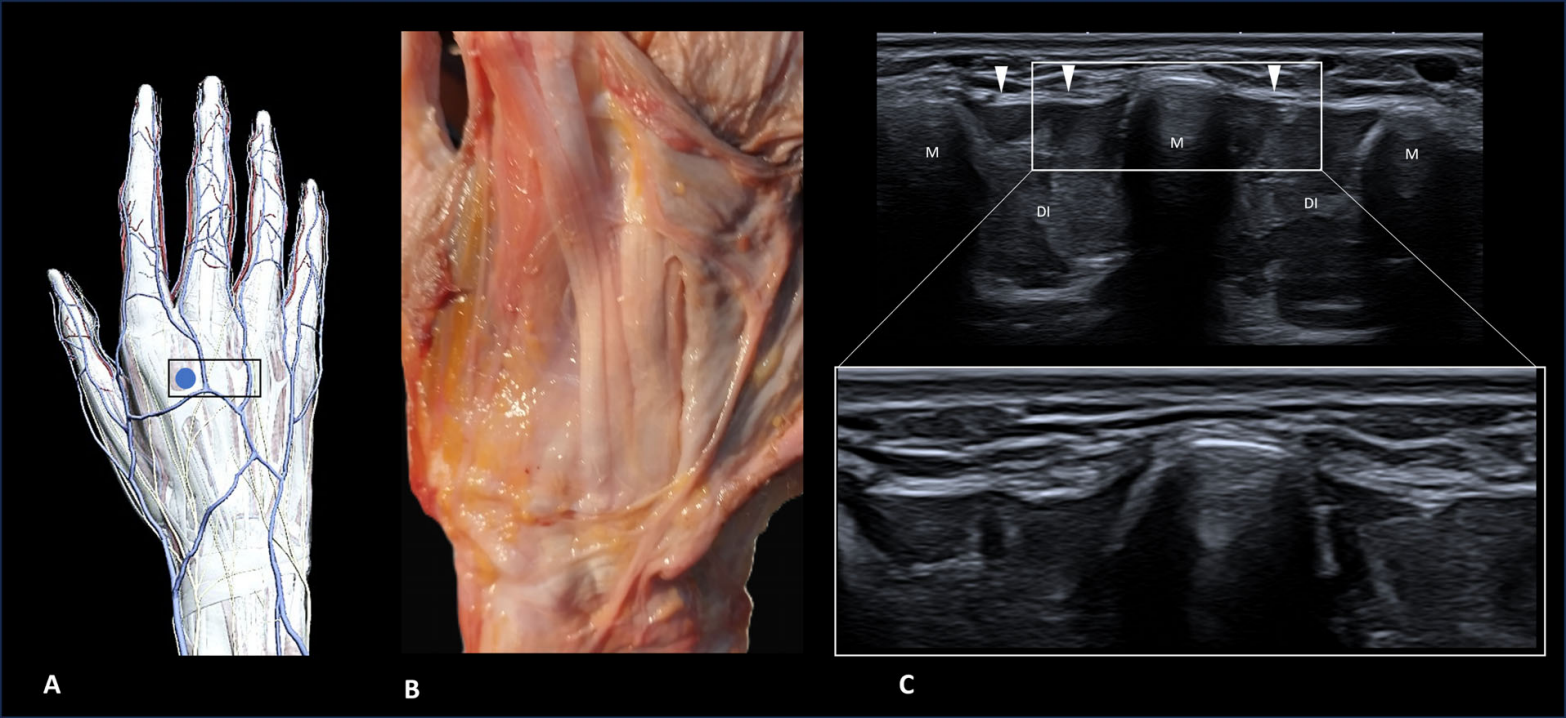
Dorsal fascia of the hand. **A**, Probe positioning. **B**, Anatomical dissection showing the dorsal fascia of the hand, appearing as epimysial layers, often less distinct from underlying muscles. **C**, Ultrasound images revealing the dorsal fascia of the hand (arrowheads) as a thin hyperechoic band. M, Metacarpal bone; DI, dorsal interossei muscles.

To perform an US examination of the dorsal fascia of the hand, the patient's hand must be in a relaxed position, resting on a flat surface with the dorsum facing upward. The fingers are slightly extended to avoid excessive tension on the fascia. The dorsal fascia is relatively thin; therefore, a small foot print (hockey‐stick probe) can be useful for scanning around the metacarpal and phalangeal regions. The probe is kept in long axis over the metacarpal region to assess the continuity of the superficial and deep lamina of the dorsal fascia. Then, short axis imaging is done to evaluate the fascial thickness and its relation to underlying structures, such as extensor tendons and interosseous muscles. The superficial lamina can be recognized as covering the extensor tendons and continuing proximally with the extensor retinaculum, and the deep as it lies above the dorsal interosseous muscle and attaches to the periosteum with extensor retinaculum. Inter‐tendinous connections should be assessed for any thickening or fibrosis. For the dynamic assessment, the patient is asked to extend and flex the fingers while scanning to evaluate the gliding of the extensor tendons within fascia (Figure 11).

#### Clinical Relevance

Fibrotic alterations or adhesions within dorsal fascia can restrict extensor tendon mobility, leading to diminished finger extension and reduced dexterity.^55^ Moreover, fascial thickening or inflammation may contribute to post‐traumatic stiffness and overuse syndromes, in particular in athletes and manual laborers, where repetitive strain exacerbates dysfunction.^56^ Given these implications, clinical applications include post‐traumatic evaluations of fascial integrity following hand injuries, assessment of dorsal hand pain related to fasciitis, nerve entrapment or extensor tendinopathy and preoperative planning for surgeries involving extensor tendons or fascial release (Table 3).^57^

### Deep Fasciae of the Fingers

#### Anatomy

They are highly specialized and play a crucial role in maintaining the mechanical efficiency and integrity of the hand during movement. Unlike other regions of the body, the fingers lack a distinct superficial fascia. Instead, the skin is directly anchored to the underlying deep fascia through a network of fibrous septa, which prevents excessive displacement of the skin during flexion and extension, ensuring precise and coordinated motion. The deep fascia of the fingers is reinforced around each joint by retinacular structures, which contribute to stability and optimal force transmission. Rayan et al^58^ described the extensor retinacular system as a complex integration of extrinsic and intrinsic musculotendinous components, comprising transverse, sagittal, and oblique fibers. The transverse‐sagittal fibers form a cylindrical sheath around the metacarpal head, creating a stable environment for tendon excursion. Sagittal bands envelop the extensor digitorum tendons and the superficial interosseus tendons bilaterally, while oblique fibers contribute to the formation of the triangular lamina distal to the sagittal band.^58^ These retinacular structures are not isolated entities but rather components of an interconnected tissue complex that meets diverse functional demands. This 3‐dimensional fibrous network acts as a structural skeleton that optimizes the mechanical performance of the fingers. Bendz^59^ emphasized the critical role of the oblique ligaments in coordinating phalangeal motion, in particular synchronizing the movements of the 2 distal phalanges and initiating the extension of a fully flexed distal phalanx.

#### Ultrasonography

To visualize the deep fascia of the fingers, a small foot print (hockey‐stick) probe can be useful for scanning around the metacarpal and phalangeal regions. The patient must be seated with the hand resting on a firm surface, palm facing upward for volar and downward for dorsal scans. Aligning the probe in long axis parallel to the finger helps to evaluate the continuity of the fascial layers. Then, short axis scanning is done by placing the probe perpendicular to the digit to assess fascial thickness and detect possible pathologies such as adhesions, fibrosis, or densification. The scanning depth must be set to 5–10 mm, depending on the finger size, to capture detailed fascial structures. Applying minimal pressure to avoid distortion of the fascial planes and ensuring accurate assessment of fascial gliding is important. Active flexion and extension of the fingers helps to evaluate fascial gliding and detect potential entrapment of tendons or nerves. Deep fascia appears as a hyperechoic band surrounding the flexor and extensor tendons. Retinacular structures are seen as thickened hyperechoic bands at each joint level, ensuring tendon stability. Interosseus and lumbrical muscle insertions can be visualized attaching to the deep fascia of fingers, especially in the volar region.

#### Clinical Relevance

US examination of the deep fasciae of the fingers plays a pivotal role in diagnosing conditions such as trigger finger, Dupuytren's contracture and post‐traumatic adhesions. Their problems can contribute to conditions such as retinacula dysfunction, tendon entrapment syndromes, and post‐traumatic adhesions, all of which impact hand performance and dexterity (Table 3).^59^

## Conclusions

The deep fasciae of the upper limb are essential for musculoskeletal force transmission and play a role in pain generation. High‐resolution US imaging, supported by anatomical and histological studies, enables a comprehensive assessment of fascial morphology and functional integrity. The standardized scanning protocols proposed in this study hold strong educational value, providing a reproducible framework for teaching and learning deep fascial sonoanatomy through direct correspondence between US imaging, gross anatomy and histology. From a clinical standpoint, this approach enhances diagnostic precision, thus guiding targeted interventional strategies and rehabilitation. Overall, these integrated protocols represent a step forward in promoting consistent fascial evaluation within research and clinical musculoskeletal US practice.

## Data Availability

The data that support the findings of this study are available on request from the corresponding author. The data are not publicly available due to privacy or ethical restrictions.

## References

[1] Pirri C , Stecco C , Fede C , Macchi V , Özçakar L . Ultrasound imaging of the fascial layers: you see (only) what you know. J Ultrasound Med 2020; 39:827–828. 10.1002/jum.15148. 31642543

[2] Langevin HM , Fox JR , Koptiuch C , et al. Reduced thoracolumbar fascia shear strain in human chronic low back pain. BMC Musculoskelet Disord 2011; 12:203. 10.1186/1471-2474-12-203. 21929806 PMC3189915

[3] Albano D , Aringhieri G , Messina C , De Flaviis L , Sconfienza LM . High‐frequency and ultra‐high frequency ultrasound: musculoskeletal imaging up to 70 MHz. Semin Musculoskelet Radiol 2020; 24:125–134. 10.1055/s-0039-3401042. 32438439

[4] Adstrum S , Hedley G , Schleip R , Stecco C , Yucesoy CA . Defining the fascial system. J Bodyw Mov Ther 2017; 21:173–177. 10.1016/j.jbmt.2016.11.003. 28167173

[5] Fede C , Pirri C , Fan C , et al. A closer look at the cellular and molecular components of the deep/muscular fasciae. Int J Mol Sci 2021; 22:1411. 10.3390/ijms22031411. 33573365 PMC7866861

[6] Iwanaga J , Singh V , Takeda S , et al. Standardized statement for the ethical use of human cadaveric tissues in anatomy research papers: recommendations from anatomical journal editors‐in‐chief. Clin Anat 2022; 35:526–528. 10.1002/ca.23849. 35218594

[7] Rispoli DM , Athwal GS , Sperling JW , Cofield RH . The anatomy of the deltoid insertion. J Shoulder Elbow Surg 2009; 18:386–390. 10.1016/j.jse.2008.10.012. 19186076

[8] Stecco C . Functional Atlas of the Human Fascial System. 1st ed. Edinburgh: Elsevier; 2015.

[9] Van de Wall BJM , Hoepelman RJ , Michelitsch C , et al. Minimally invasive plate osteosynthesis (MIPO) for scapular fractures. Oper Orthop Traumatol 2023; 35:390–396. 10.1007/s00064-023-00819-5. 37594566 PMC10697868

[10] Bhansing KJ , Van Rosmalen MH , Van Engelen BG , Vonk MC , Van Riel PL , Pillen S . Increased fascial thickness of the deltoid muscle in dermatomyositis and polymyositis: an ultrasound study. Muscle Nerve 2015; 52:534–539. 10.1002/mus.24595. 25655014

[11] Boussaa H , Kamoun M , Miladi S , et al. The first case of SARS‐CoV‐2‐induced eosinophilic fasciitis. Mod Rheumatol Case Rep 2023; 8:224–228. 10.1093/mrcr/rxad063. 37902576

[12] Stecco A , Pirri C , Stecco C . Fascial entrapment neuropathy. Clin Anat 2019; 32:883–890. 10.1002/ca.23388. 31004463

[13] Khan A , Chakravorty A , Gui GP . In vivo study of the surgical anatomy of the axilla. Br J Surg 2012; 99:871–877. 10.1002/bjs.8737. 22505319

[14] Georgiev GP , Tubbs RS . What is the axillary arch (of Langer)? J Anat 2024; 245:197–198. 10.1111/joa.14037. 38444373 PMC11161814

[15] Kim PW . Variations of the musculofascial axillary arch with the adjacent lymph nodes. Surg Radiol Anat 2021; 43:27–32. 10.1007/s00276-020-02544-1. 32804254

[16] Takeishi M , Ishida K , Makino Y . The thoracodorsal vascular tree‐based combined fascial flaps. Microsurgery 2009; 29:95–100. 10.1002/micr.20579. 18942653

[17] Beylacq L , Baer E , Choquet O , Dupre HL , Capdevila X . Perifascial plane versus perineural approaches for ultrasound‐guided axillary block: go to the simplest? Minerva Anestesiol 2020; 86:23–29. 10.23736/S0375-9393.19.12402-9. 31630504

[18] Cocco G , Ricci V , Ricci C , et al. Ultrasound imaging of the axilla. Insights Imaging 2023; 14:78. 10.1186/s13244-023-01430-9. 37166516 PMC10175532

[19] Borg MB , Mittino L , Battaglia M , et al. Tolerability, safety and efficacy of a specific rehabilitation treatment protocol for axillary web syndrome: an observational retrospective study. Cancers (Basel) 2023; 15:426. 10.3390/cancers15020426. 36672375 PMC9856526

[20] Meer TA , Noor R , Bashir MS , Ikram M . Comparative effects of lymphatic drainage and soft tissue mobilization on pain threshold, shoulder mobility and quality of life in patients with axillary web syndrome after mastectomy. BMC Womens Health 2023; 23:588. 10.1186/s12905-023-02762-w. 37950230 PMC10638722

[21] Singer E . Fasciae of the Human Body and their Relations to the Organs they Envelop. Baltimore: Williams & Wilkinns Company; 1935:19‐21.

[22] Zappia M , Ascione F , Romano AM , et al. Comma sign of subscapularis tear: diagnostic performance and magnetic resonance imaging appearance. J Shoulder Elbow Surg 2021; 30:1107–1116. 10.1016/j.jse.2020.07.047. 32835804

[23] Moccia D , Nackashi AA , Schilling R , Ward PJ . Fascial bundles of the infraspinatus fascia: anatomy, function, and clinical considerations. J Anat 2016; 228:176–183. 10.1111/joa.12386. 26403802 PMC4694164

[24] Chafik D , Galatz LM , Keener JD , Kim HM , Yamaguchi K . Teres minor muscle and related anatomy. J Shoulder Elbow Surg 2013; 22:108–114. 10.1016/j.jse.2011.12.005. 22521388

[25] Friend J , Francis S , McCulloch J , Ecker J , Breidahl W , McMenamin P . Teres minor innervation in the context of isolated muscle atrophy. Surg Radiol Anat 2010; 32:243–249. 10.1007/s00276-009-0605-9. 20020125

[26] Ricci V , Chang KV , Güvener O , et al. EURO‐MUSCULUS/USPRM dynamic ultrasound protocols for shoulder. Am J Phys Med Rehabil 2022; 101:e29–e36. 10.1097/PHM.0000000000001833. 34923500

[27] Pirri C , Pirri N , Stecco C , et al. Sono‐palpation and sono‐Tinel in musculoskeletal ultrasound examination: use all “sono‐senses”. A systematic review. Med Ultrason 2023; 25:325–329. 10.11152/mu-4060. 37369030

[28] Moraiti K , Zampeli F , Reinares F , Gantsos A , Valenti P . Feasibility of lower trapezius transfer extended by the infraspinatus fascia for restoration of external rotation in irreparable posterosuperior rotator cuff tears: an anatomical study. Eur J Orthop Surg Traumatol 2021; 31:661–667. 10.1007/s00590-020-02817-w. 33098460

[29] Duparc F , Coquerel D , Ozeel J , Noyon M , Gerometta A , Michot C . Anatomical basis of the suprascapular nerve entrapment, and clinical relevance of the supraspinatus fascia. Surg Radiol Anat 2010; 32:277–284. 10.1007/s00276-010-0631-7. 20309668

[30] Al‐Redouan A , Holding K , Kachlik D . “Suprascapular canal”: anatomical and topographical description and its clinical implication in entrapment syndrome. Ann Anat 2021; 233:151593. 10.1016/j.aanat.2020.151593. 32898658

[31] Singer E . Fascia of the Human Body and Their Relations to the Organs They Envelop. Philadephia: Williams and Wilkins; 1935.

[32] Bektas U , Ay S , Yilmaz C , Tekdemir I , Elhan A . Spinoglenoid septum: a new anatomic finding. J Shoulder Elbow Surg 2003; 12:491–492. 10.1016/s1058-2746(03)00065-x. 14564274

[33] Stecco A , Macchi V , Stecco C , et al. Anatomical study of myofascial continuity in the anterior region of the upper limb. J Bodyw Mov Ther 2009; 13:53–62. 10.1016/j.jbmt.2007.04.009. 19118793

[34] Stecco C , Gagey O , Macchi V , et al. Tendinous muscular insertions onto the deep fascia of the upper limb. First part: anatomical study. Morphologie 2007; 91:29–37. 10.1016/j.morpho.2007.05.001. 17574470

[35] Stecco C , Porzionato A , Macchi V , et al. The expansions of the pectoral girdle muscles onto the brachial fascia: morphological aspects and spatial disposition. Cells Tissues Organs 2008; 188:320–329. 10.1159/000121433. 18349526

[36] Pirri C , Guidolin D , Fede C , Macchi V , De Caro R , Stecco C . Ultrasound imaging of brachial and antebrachial fasciae. Diagnostics (Basel) 2021; 11:2261. 10.3390/diagnostics11122261. 34943498 PMC8700752

[37] Windisch G , Tesch NP , Grechenig W , Peicha G . The triceps brachii muscle and its insertion on the olecranon. Med Sci Monit 2006; 12:BR290–BR294. 16865062

[38] Keener JD , Chafik D , Kim HM , Galatz LM , Yamaguchi K . Insertional anatomy of the triceps brachii tendon. J Shoulder Elbow Surg 2010; 19:399–405. 10.1016/j.jse.2009.10.008. 20056450

[39] van der Wal J . The architecture of the connective tissue in the musculoskeletal system‐an often overlooked functional parameter as to proprioception in the locomotor apparatus. Int J Ther Massage Bodywork 2009; 2:9–23. 10.3822/ijtmb.v2i4.62. 21589740 PMC3091473

[40] Amado FC , Noversa C , Moura A , Carvalho L , Cardoso L . Necrotizing fasciitis causing acute compartment syndrome after radial artery catheterization. Eur J Case Rep Intern Med 2020; 7:001525. 10.12890/2020_001525. 32309262 PMC7162573

[41] Titolo P , Milani P , Panero B , Ciclamini D , Colzani G , Artiaco S . Acute compartment syndrome of the arm after minor trauma in a patient with optimal range of oral anticoagulant therapy: a case report. Case Rep Orthop 2014; 2014:980940. 10.1155/2014/980940. 24516765 PMC3912892

[42] Mai MC , Beck R , Gabriel K , Singh KA . Posterior arm compartment syndrome after a combined supracondylar humeral and capitellar fractures in an adolescent: a case report. J Pediatr Orthop 2011; 31:e16–e19. 10.1097/BPO.0b013e31820fc8c9. 21415676

[43] Konstantakos EK , Dalstrom DJ , Nelles ME , Laughlin RT , Prayson MJ . Diagnosis and management of extremity compartment syndromes: an orthopaedic perspective. Am Surg 2007; 73:1199–1209. 18186372

[44] Fung DA , Frey S , Grossman RB . Rare case of upper arm compartment syndrome following biceps tendon rupture. Orthopedics 2008; 31:494. 10.3928/01477447-20080501-09. 19292309

[45] Stecco C , Macchi V , Lancerotto L , Tiengo C , Porzionato A , De Caro R . Comparison of transverse carpal ligament and flexor retinaculum terminology for the wrist. J Hand Surg Am 2010; 35:746–753. 10.1016/j.jhsa.2010.01.031. 20346594

[46] Ortiz‐Miguel S , Miguel‐Pérez M , Navarro J , et al. Compartments of the antebrachial fascia of the forearm: clinically relevant ultrasound, anatomical and histological findings. Surg Radiol Anat 2021; 43:1569–1579. 10.1007/s00276-021-02736-3. 33818623

[47] Tao KZ , Chen EY , Ji RM , Dang RS . Anatomical study on arteries of fasciae in the forearm fasciocutaneous flap. Clin Anat 2000; 13:1–5. 10.1002/(SICI)1098-2353(2000)13:1<1::AID-CA1>3.0.CO;2-6. 10617882

[48] Fede C , Coldebella L , Petrelli L , Bassetto F , Tiengo C , Stecco C . Biochemical and histological differences between longitudinal and vertical fibres of Dupuytren's palmar aponeurosis and innovative clinical implications. Int J Mol Sci 2024; 25:6865. 10.3390/ijms25136865. 38999972 PMC11241458

[49] Legueu F , Juvara E . Des aponévroses de la paume de la main. Bull Soc Anat Paris 1892; 67:383–400.

[50] Ling MZX , Kumar VP . Myofascial compartments of the hand in relation to compartment syndrome: a cadaveric study. Plast Reconstr Surg 2009; 123:613–616. 10.1097/PRS.0b013e3181956538. 19182620

[51] Kondrup F , Gaudreault N , Venne G . The deep fascia and its role in chronic pain and pathological conditions: a review. Clin Anat 2022; 35:649–659. 10.1002/ca.23882. 35417568

[52] Lee K , Park JM , Yoon SY , et al. Ultrasound‐guided nerve hydrodissection for the management of carpal tunnel syndrome: a systematic review and network meta‐analysis. Yonsei Med J 2025; 66:111–120. 10.3349/ymj.2024.0089. 39894044 PMC11790405

[53] Rotman MB , Donovan JP . Practical anatomy of the carpal tunnel. Hand Clin 2002; 18:219–230. 10.1016/s0749-0712(01)00003-8. 12371025

[54] Landsmeer JM . The anatomy of the dorsal aponeurosis of the human finger and its functional significance. Anat Rec 1949; 104:31–44. 18149912 10.1002/ar.1091040105

[55] Jia Q , Chen D , Guo J , et al. Risk factors associated with tendon adhesions after hand tendon repair. Front Surg 2023; 10:1121892. 10.3389/fsurg.2023.1121892. 37143766 PMC10151704

[56] Verdon ME . Overuse syndromes of the hand and wrist. Prim Care 1996; 23:305–319. 10.1016/s0095-4543(05)70278-5. 8784932

[57] Xiao G , Wang J , Zhang N , Hao J . Factors predicting the adhesion and prolonged lost days of work in patients with extensor tendon adhesion of the hand. Front Surg 2024; 11:1304202. 10.3389/fsurg.2024.1304202. 38752129 PMC11094267

[58] Rayan GM , Murray D , Chung KW , Rohrer M . The extensor retinacular system at the metacarpophalangeal joint. Anatomical and histological study. J Hand Surg Br 1997; 22:585–590. 10.1016/s0266-7681(97)80351-8. 9752909

[59] Bendz P . The functional significance of the oblique retinacular ligament of Landsmeer. A review and new proposals. J Hand Surg Br 1985; 10:25–29. 10.1016/s0266-7681(85)80009-7. 3998597

